# Effects of Protein Supplementation Combined with Exercise Intervention on Frailty Indices, Body Composition, and Physical Function in Frail Older Adults

**DOI:** 10.3390/nu10121916

**Published:** 2018-12-04

**Authors:** Chun-De Liao, Pi-Hsia Lee, Dun-Jen Hsiao, Shih-Wei Huang, Jau-Yih Tsauo, Hung-Chou Chen, Tsan-Hon Liou

**Affiliations:** 1 School and Graduate Institute of Physical Therapy, College of Medicine, National Taiwan University, Taipei 10055, Taiwan; 08415@s.tmu.edu.tw (C.-D.L.); jytsauo@ntu.edu.tw (J.-Y.T.); 2 Department of Physical Medicine and Rehabilitation, Shuang Ho Hospital, Taipei Medical University, Taipei 23561, Taiwan; 13001@s.tmu.edu.tw (S.-W.H.); 10462@s.tmu.edu.tw (H.-C.C.); 3 School of Nursing, College of Nursing, Taipei Medical University, Taipei 10675, Taiwan; pihsia@tmu.edu.tw; 4 School and Graduate Institute of Nutrition Science, College of Medicine, Taipei Medical University, Taipei 23561, Taiwan; antifat53@gmail.com; 5 Graduate Institute of Sports Science, National Taiwan Sport University, Taoyuan 33371, Taiwan; 6 Center for Evidence-Based Health Care, Shuang Ho Hospital, Taipei Medical University, Taipei 23561, Taiwan; 7 Department of Physical Medicine and Rehabilitation, School of Medicine, College of Medicine, Taipei Medical University, Taipei 23561, Taiwan; 8 Obesity Research Center, Wan Fang Hospital, Taipei Medical University, Taipei 23561, Taiwan

**Keywords:** frailty, protein supplementation, exercise training, lean body mass, physical function

## Abstract

Aging poses a high risk of lean mass loss, which can be effectively improved through resistance exercise training (RET), or multicomponent exercise training (MET) as well as nutrition supplementation, such as protein supplementation (PS). This study investigated the effects of PS plus exercise training on frail older individuals. A comprehensive search of online databases was performed to identify randomized controlled trials (RCTs) that reported the efficacy of PS combined with RET or MET in frail older individuals. The included RCTs were analyzed through a meta-analysis and risk-of-bias assessment. We finally included 22 RCTs in the meta-analysis, with a mean (range/total) Physiotherapy Evidence Database score of 6.7 (4–9/10). PS plus exercise training significantly improved the frailty status (odds ratio = 2.77; *p* = 0.006), lean mass (standard mean difference (SMD) = 0.52; *p* < 0.00001), leg strength (SMD = 0.37; *p* < 0.00001), and walking speed (SMD = 0.32; *p* = 0.002). Subgroup analyses revealed that PS plus MET exert significant effects on frailty indices, whereas PS plus RET further improves lean mass. Our findings suggest that PS plus RET as well as MET is effective in improving frailty status, lean mass, muscle strength, and physical mobility in frail older individuals.

## 1. Introduction

Frailty, characterized by muscle weakness and slow walking speed, has been considered to be closely associated with sarcopenia [1,2]. Aging-associated decline in muscle mass contributes to the related low physical performance in older adults [3,4,5]. Because frail persons, especially institutionalized residents, are at a high risk of undernutrition [6] and physical inactivity [7,8], identifying prompt nutrition and exercise interventions to reserve and maintain muscle mass in frail older adults is crucial.

Based on the multifactorial genesis of frailty, an interdisciplinary approach combining nutrition and exercise interventions has been advocated to counteract the complexity of pathogenesis of such geriatric syndromes [2,9]. Exercise interventions, such as resistance exercise training (RET) that is employed alone or comprises multicomponent exercise training (MET)—including aerobics, balance, and function tasks—have been used as an effective method for improving muscle function and increasing muscle mass by stimulating muscle protein synthesis in older adults [10,11,12,13,14]. However, muscle hypertrophy induced by RET highly depends on the training intensity that is generally not tolerated and afforded by frail individuals. Therefore, incorporating RET into MET is advised as a viable exercise type that can be easily adapted by frail individuals [15,16].

Recent systematic reviews have summarized the synergistic effects of PS and ET on body composition and strength in older individuals [15,17,18,19,20], most of which included healthy participants [18,19,20,21] and included trials using RET only [18,19,20,21]. In addition, few of the aforementioned systematic reviews focused on the effects of PS plus MET in frail older adults. Therefore, this study analyzed the effect of PS plus RET or MET on the frailty status of older adults. Through subgroup analyses, we also identified the difference in effects on body composition and physical outcomes between RET and MET.

## 2. Method

### 2.1. Design

The present study was conducted by following the guidelines recommended by the Preferred Reporting Items for Systematic Reviews and Meta-Analysis [22]. The protocol for this study was registered at PROSPERO (registration number: CRD42018109176). The study was conducted on the basis of a comprehensive electronic search from online sources. Articles were obtained from online databases, namely PubMed, EMBASE, the Cochrane Library Database, the Physiotherapy Evidence Database (PEDro), China Knowledge Resource Integrated Database, and Google Scholar. Secondary sources included papers cited by articles retrieved from the abovementioned sources. No limitation was imposed on the publication year and language to minimize publication and language bias. Two reviewers (C.D.L. and H.C.C.) independently searched for articles, screened studies, and extracted data. Any disagreements between the reviewers were resolved through consensus, with other team members (T.H.L. and D.J.H.) acting as arbiters.

### 2.2. Search Strategy

The following keywords for participant conditions were used: “older/aging/aged/elder/seniors” and “frailty/frail”.

The keywords used for intervention were as follows: “progressive resistance training, resistance exercise, strength training, weight training and/or weight lifting”; “multicomponent exercise, physical activity exercise”; and “protein/amino-acid/nutrient supplement”. The detailed search formulas used for each database are presented in online Table S1.

### 2.3. Study Selection Criteria

Studies were included if they met the following criteria: (1) the study was a randomized controlled trial (RCT); (2) experimental groups received PS (including adequate protein-based diet) plus exercise intervention (i.e., RET or MET); (3) control groups received a placebo supplement, PS alone, exercise training alone, or none of the above; (4) the supplement intervention involved protein sources including whey protein; leucine; casein; soy; and bovine colostrum isolate, concentrate, or hydrolysate consumed in isolation or in combination with other nutrients (creatine and amino acids); (5) participants were institutionalized residents or community-dwelling elders older than 60 years and were at risk of becoming frail; (6) the study reported the outcome measures including primary or secondary outcomes that were defined below.

Articles were eliminated if any of the following exclusion criteria were met: (1) the trial enrolled pre-frail older individuals; (2) the article reported a study that was conducted in vitro or vivo with an animal model; and (3) the article had a non-RCT design, such as a case report, case series, or a prospectively designed trial without a comparison group.

### 2.4. Data Extraction

Data on the following were extracted from each included study and presented in an evidence table (Table 1): (1) characteristics of the study design and sample (population area, group design, sex, age, body mass index, and patient type); (2) characteristics of exercise training and PS; (3) measured time points; and (4) main outcome results. One author (C.D.L.) extracted relevant data from included studies, and another author (S.W.H.) reviewed the extracted data. Any disagreement between the two authors was resolved through consensus. A third author (T.H.L.) was further consulted if the disagreement persisted.

The trial parallels with the PS plus exercise training group were extracted as experimental groups and those with placebo supplement, PS alone, or exercise training alone were extracted as control groups. If the trial had more than one experimental group or control intervention, we combined multiple experimental or control groups to create a single pair-wise comparison for meta-analyses which is recommended in Cochrane’s Handbook [23].

### 2.5. Outcome Measures

The primary outcomes in this study included frailty indices and body composition. The frailty indices used in this study were derived from the Fried’s frailty criteria which comprises five components including whole body mass, handgrip strength, walking speed, exhaustion, and physical activity [24]; a global frailty score was defined as the number of five Fried’s frailty components. Body weight or body mass index were extracted for meta-analyses. The measures used to assess gait speed such as walking on a set distance (6 m, 10 m, etc.) and six-minute walk test were extracted and meta-analyzed to identify walking speed outcome. The measures used to assess fatigue and engagement in social activity, such as the 36-Item Short Form Health Survey vitality subscale [25] and the Minimum Data Set social engagement subscale [26], were extracted and meta-analyzed to identify exhaustion outcome. Body composition measures included lean body mass, fat-free mass, appendicular lean mass, and fat mass. The secondary outcomes were physical function included leg strength, mobility (i.e., chair rise, timed up-and-go, and short physical performance battery), and ADLs. Negative score changes of the measures (e.g., timed up-and-go) which denoted improved effects were transformed to positive values for meta-analyses.

### 2.6. Assessment of Bias Risks and Methodological Quality of Included Studies

The quality of the included trials was assessed using the PEDro quality score to assess the risk of bias. The methodological quality of all the included studies was independently assessed by two researchers in accordance with the PEDro classification scale, which is a valid measure of the methodological quality of clinical trials [27]. The PEDro scale scores 10 items, namely random allocation, concealed allocation, similarity at baseline, subject blinding, therapist blinding, assessor blinding, >85% follow-up for at least one key outcome, intention-to-treat analysis, between-group statistical comparison for at least one key outcome, and point and variability measures for at least one key outcome. Each item is scored as either 1 for present or 0 for absent, and a total score ranging from 0 to 10 is obtained through summation of all the 10 items. An interrater reliability generalized kappa statistic between 0.40 and 0.75 was reported for the PEDro scale [28], and an intraclass correlation coefficient associated with the cumulative PEDro score of 0.91 (95% confidence interval (CI): 0.84–0.95) was reported for nonpharmacological studies [29]. On the basis of the PEDro score, the methodological quality of the included RCTs was rated as high (≥7/10), medium (4–6/10), and low (≤3/10) [30].

We graded the levels of evidence (LoE) for each outcome of interest according to the guideline of evidence synthesis [31] derived from the criteria of van Tulder [32] (Table S2).

### 2.7. Data Synthesis and Analysis

We computed effect sizes for each study separately for primary and secondary outcome measures. Primary outcomes, namely body composition, muscle strength, and muscle structure, were defined as a pooled estimate of the mean difference in changes between the treatment (PS and resistance training) and placebo (other type of supplement and resistance training) groups. If the exact variance of the paired difference was not derivable, it was imputed by assuming a correlation coefficient of 0.98 between the baseline and posttest measured data [33,34]. If data were reported as the median (range), they were recalculated algebraically from the trial data to impute the sample mean and SD [23,35]. All the extracted outcome data were calculated as the standard mean difference (SMD) versus placebo or active control as well as secondary outcomes including functional mobility. We used SMD for meta-analysis when different scales were used to measure the same concept (e.g., walking speed and function score). We categorized the magnitude of SMD in accordance with the following version of Cohen’s criteria [36]: trivial (*d* < 0.10), small (0.10 ≤ *d* < 0.25), medium (0.25 ≤ *d* < 0.40), and large (*d* ≥ 0.40).

Fixed-effect or random-effect models were used depending on the existence of heterogeneity. Statistical heterogeneity was assessed using the *I*^2^ statistic and was estimated for significance (*p* < 0.05) and χ² and F values greater than 50% [37]. A fixed-effects model was used unless statistical heterogeneity was significant (*p* < 0.05), and subsequently, a random-effects model was used.

The duration of follow up was assessed and defined as short term (<3 months), medium term (≥3 and <6 months), and long term (≥6 and <12 months).

Subgroup analysis was conducted by considering the methodological quality level, participant type (i.e., community dweller or institutionalized resident), population area, type of the control group (i.e., placebo, PS alone, or exercise training alone), exercise type (i.e., RET or MET), supplementation dose (i.e., high-dose and low-dose), and duration of intervention in the included trials. Based on that, a sufficient PS up to 30–40 g after RET has been indicated to augment the effects of resistance training on muscle mass gain in older adults [38], a cut off value of 30 g of PS per day or per exercise session was used to define high and low dose of supplementation. All subgroup differences were tested for significance, and an *I*^2^ statistics statistic was computed to estimate the degree of subgroup variability. Potential publication bias was investigated using visual inspection of a funnel plot to explore possible reporting bias [39] for primary outcome measures which had clinically relevant results, and was assessed using the Egger’s regression asymmetry test [40] with the SPSS, Version 17.0, statistical software (IBM, Armonk, NY, USA). A *P* value of <0.05 was considered statistically significant. All analyses were conducted using RevMan 5.3 (The Nordic Cochrane Centre, Copenhagen, Denmark).

## 3. Results

### 3.1. Trial Flow

**Figure 1:**
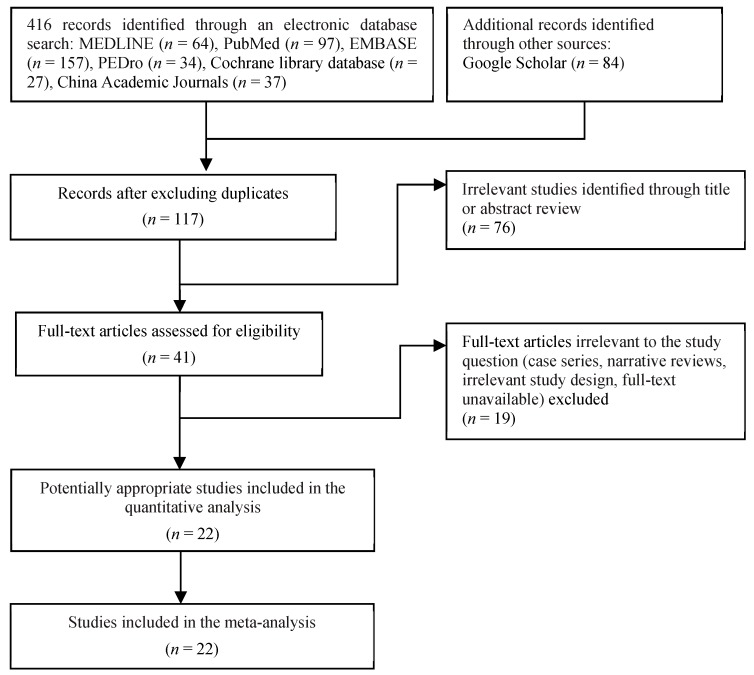
Flowchart of the study selection process.

Figure 1 shows a flow diagram of the selection process. The final sample consisted of 22 RCTs [26,41,42,43,44,45,46,47,48,49,50,51,52,53,54,55,56,57,58,59,60,61] published from 1994 to 2017. A total sample of 2113 (1525 women) frail older participants with a mean (SD) age of 82.3 (5.1) years was enrolled. Of all participants, 750 (544 women) received a protein-type supplement in combination with exercise training, 610 (424 women) received exercise training alone, 267 (204 women) received PS alone, and 486 (353 women) received a placebo supplement.

### 3.2. Study Characteristics

Table 1 summarizes the demographic data and study characteristics of the included RCTs. Eleven RCTs enrolled participants who were community-dwelling frail elders [43,44,45,46,47,48,52,54,58,60,61], whereas the other 11 enrolled institutionalized residents [26,41,42,49,50,51,53,55,56,57,59]; population areas included Americas (three RCTs) [46,49,55], Asia (four RCTs) [52,53,54,60], Europe (14 RCTs) [26,41,42,43,44,45,47,48,51,56,57,58,59,61], and Oceania (one RCT) [50]. Regarding the duration of intervention, most of the included RCTs employed an intervention period of three to six months [44,45,46,47,48,50,51,52,53,54,55,56,57,58,59,60]; another five RCTs performed a short intervention period less than three months [26,41,42,49,61]; and one had a long period of nine months [43]. With respect to the follow-up duration, all the 22 included RCTs reported a short-term or medium outcome of less than six months; 14 RCTs had a long-term follow-up to nine months [26,42,43,44,46,48,50,51,53,54,56,57,58,60].

### 3.3. Protein Supplementation Characteristics

Protocols for PS are summarized in Table 1, and the supplement program of each included RCT is detailed in Table S3. The protocol for PS varied widely across the included trials. Regarding the amount of protein, the majority of the included RCTs provided PS daily with amounts of extra protein ranging from 4.1 to 40.8 g/day [26,42,43,48,49,50,51,53,54,55,56,58,59,60,61]. Twelve RCTs provided supplements before or after exercise on training days with amounts of extra protein ranging from 6.0 to 41.4 g/session [26,41,42,44,46,50,51,52,54,56,57,61].

The protein source of the supplementation differed among the included studies and comprised milk-based beverages, fortified milk, and milk protein concentrate [26,42,44,48,54,57,58]; a combination of whey protein, leucine, and essential amino acids [50,51,52,55,56,59,60]; and dairy through diet [45,47,49,53]. Supplements were provided daily in most included RCTs [26,42,43,45,47,48,49,50,51,53,54,55,56,58,59,60,61], whereas 12 RCTs provided an additional PS immediately before or after exercise on training days [26,41,42,44,46,50,51,52,54,56,57,61]. In 10 RCTs, the control group received a placebo supplement or diet [43,44,45,48,49,52,54,58,59,61], and in the remaining 12 included RCTs, the control group was subjected to exercise sessions only without placebo supplement intake [26,41,42,46,47,50,51,53,55,56,57,60] (Table S3). Fourteen of the 22 the included RCTs reported compliance for PS in their participants, most of which reported well compliance for supplementations (80–100%) [26,41,42,44,45,46,49,52,57,58,59,60,61], with the exception of one RCT reported a low compliance rate of 61% [43].

### 3.4. Exercise Training Protocol

A summary of protocols for exercise training is presented in Table 1, and the exercise regime of each included RCT is detailed in Table S4. Participants in six RCTs received RET only [48,49,50,51,56,58] with an intensity of 50% to 80% of one repetition maximum (or OMNI-Resistance Exercise Scale > 7), whereas those in other 15 RCTs received MET with moderate to high intensity [42,43,44,45,46,47,52,53,54,55,57,59,61]; the remaining one RCT which employed a pedometer-based walking program for the frail community-dwelling older adults was categorized as MET subgroup [60]. The MET were composed of resistance training [26,41,42,43,44,45,46,47,52,53,54,55,57,59,61], aerobic training [45,46,47,52,55,60,61], balance training [26,41,42,43,44,45,46,52,53,54,55,57,59,61], and functional mobility training [41,43,44,45,47,52,53,54,55,57,60,61]. Eight RCTs used a long-period exercise duration of 24 weeks or longer (48–168 sessions) [43,46,48,50,51,56,58,60], eight RCTs used a medium-period treatment duration of 12–24 weeks (24–48 sessions) [44,45,47,52,53,54,57,59], and the other six RCTs used a short-period intervention of less than 12 weeks (22–35 sessions) [26,41,42,49,55,61]. Thirteen RCTs involved whole-body training (upper and lower extremities and trunk) [26,41,42,43,48,50,51,53,54,55,56,58,60], whereas the other nine involved training of only lower extremity (Table S4) [45,46,47,49,52,57,59,61]. Compliance to RET was reported with an attendance rate of 84–100% and 71–97% in frail community-dwellers [48,58,61] and nursing-home residents [49,51,56], respectively; the attendance rates responding to MET were reported as 63–100% and 71–100% in frail community-dwellers [43,44,45,52,61] and nursing-home residents [26,41,42,46,57,59], respectively; 6 of 22 included RCTs did not reported compliance to exercise intervention [47,50,53,54,55,60].

### 3.5. Risk of Bias in Included Studies

The individual PEDro scores are listed in Table 2. Of the 22 included RCTs, the methodological quality of 11 was classified as high [43,44,45,48,49,52,54,55,57,58,61] and that of the other 11 as medium [26,41,42,46,47,50,51,53,56,59,60], with a median (range) PEDro score of 7/10 (4/10 to 9/10). The interrater reliability associated with the cumulative PEDro score was acceptable with an intraclass correlation coefficient of 0.96 (95% CI: 0.93–0.99). Of the 22 included RCTs, all incorporated random allocation, similarity at the baseline, between-group comparisons, and point estimates and variability; in addition, five incorporated concealed allocation, 11 incorporated subject blinding, 6 incorporated therapist blinding, 10 incorporated assessor blinding, 14 incorporated adequate follow up, and 14 incorporated intention-to-treat analysis.

### 3.6. Success or Improvement Rates

**Figure 2:**
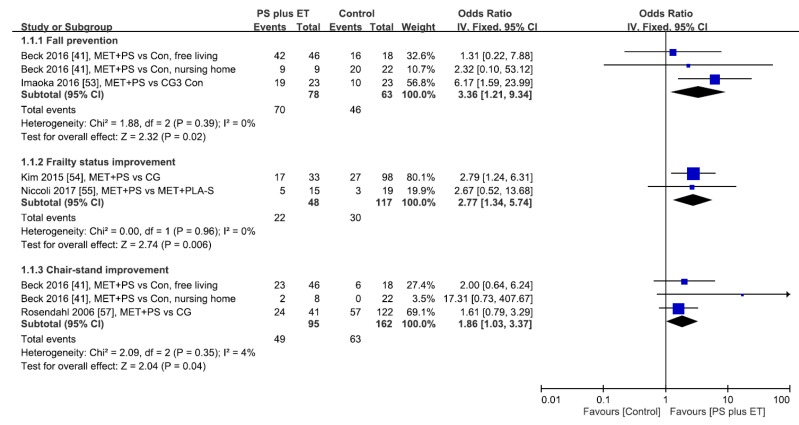
Forest plot of treatment success rates of fall prevention, frailty mitigation, and chair-stand improvement during the overall follow-up period. Each study result is represented as a point estimate (square box) with 95% CI (horizontal line). The study results plotted on the right-hand side indicate effects in favor of protein supplementation (PS) plus exercise training (ET), and the combined effects are plotted as black diamonds. 95% CI = 95% confidence interval; IV = inverse variance; Fixed = fixed-effects model; CG = control group; Con = control; MET = multicomponent exercise training; PLA-S, placebo supplement.

Categorical data for fall events [41,53], reduction in frailty status [54,55], and improvement in chair-stand task were reported (Table 1) [41,57]. The treatment success rates for fall prevention, frailty status improvement, and chair-stand improvement were meta-analyzed. The results revealed that PS accompanied with exercise intervention yielded higher treatment success rates than did the controlled comparisons in reducing fall events (LoE, moderate; OR: 3.36, 95% CI: 1.21–9.34, *P* = 0.02; *I*^2^ = 0%), diminishing frailty status (LoE, strong; OR: 2.77, 95% CI: 1.34–5.74, *P* = 0.006; *I*^2^ = 0%), and improving chair-stand performance (LoE, moderate; OR: 1.86, 95% CI: 1.03–3.37, *P* = 0.04; *I*^2^ = 4%), regardless of the follow-up duration, participant type, exercise type, and control type (Figure 2).

### 3.7. Effects on Frailty Indices

**Figure 3:**
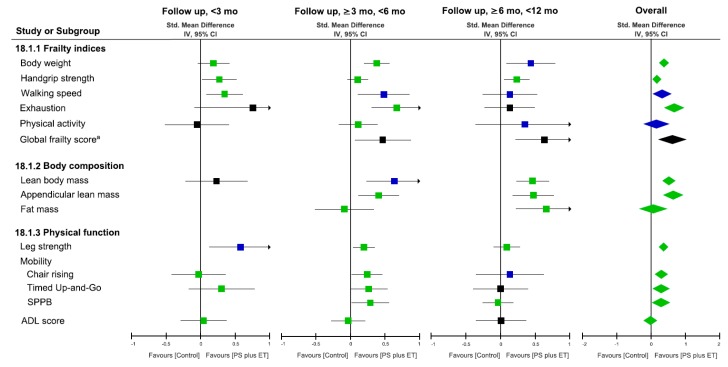
Forest plot of effects of protein supplementation (PS) plus exercise training (ET) on changes in frailty indices, body composition, and physical function at each follow-up duration. Each point estimate at each follow-up duration (square) and during the overall duration (diamond) presents the combined effect (standard mean difference) of the outcome measure where indicated, with 95% CI (horizontal line). Results plotted on the right-hand side indicate effects in favor of PS plus ET. The combined effects analyzed using a fixed- or random-effects model are denoted by green and blue colors, respectively; a black square or diamond denotes that the combined effect is derived from a single study. ^a^ Global frailty score is defined as a number out of five frailty components of Fried’s criteria [24]. 95% CI = 95% confidence interval; Std = standard; IV = inverse variance; ADL = activity of daily life; SPPB = short physical performance battery.

The effects of PS plus exercise training on the frailty indices at each follow-up duration are shown in Figure 3. Changes in whole body mass were reported by 10 RCTs [26,41,43,44,47,48,49,54,58,59], handgrip strength was reported by 13 RCTs [26,41,45,46,48,50,51,52,53,54,55,56,58], walking speed was reported by 13 RCTs [43,45,46,48,49,50,54,55,56,57,58,59,61], exhaustion was reported by two RCTs [42,59], and physical activity was reported by five RCTs [46,49,52,54,56]. In addition, one RCT reported changes in global frailty scores [54]. Generally, significant effects in favor of PS plus exercise training on frailty indices were noted at all follow-up durations, except that conflict effects could be identified in physical activity during all follow-up durations. Strong evidence suggested an overall effect of PS plus exercise training on whole body mass, with a significant SMD of 0.38 (95% CI: 0.23–0.52, *p* < 0.00001; *I*^2^ = 37%) (Figure 3 and Figure S1); similar results were observed for the effects of PS plus exercise on handgrip strength, walking speed, and exhaustion, with significant SMDs of 0.17 (95% CI: 0.05–0.30, *p* = 0.006; *I*^2^ = 26%; LoE, strong), 0.32 (95% CI: 0.05–0.59, *p* = 0.02; *I*^2^ = 75%; LoE, moderate), and 0.68 (95% CI: 0.35–1.02, *p* < 0.0001; *I*^2^ = 0%; LoE, moderate), respectively (Figure 3 and Figures S2–S6).

The results of subgroup analyses (Table 3) showed that study methodological quality, participant type, population area, control type, supplementation dose, exercise training type, and intervention duration had no effect on subgroup heterogeneity for all frailty indices (all *p* > 0.05), except that significant subgroup differences in heterogeneity were observed between different supplementation doses and intervention periods for whole body mass (*p* = 0.03, *I*^2^ = 78.2%) and walking speed (*p* = 0.006, *I*^2^ = 80.8%), respectively.

### 3.8. Effects on Body Composition

Changes in lean body mass or fat-free mass after PS plus exercise training were reported by seven RCTs [43,44,48,49,53,58,60], and changes in appendicular lean mass were reported by three RCTs [48,54,58] (Table 1). The results of meta-analyses showed significant medium-term (SMD 0.64, *p* = 0.003; LoE, moderate) and long-term (SMD 0.51, *p* = 0.002; LoE, strong) effects of PS plus exercise training on lean body mass as well as appendicular lean mass (Figure 3 and Figures S7–S8). Strong evidence suggests an overall effect of PS plus exercise training on lean body mass, with a significant SMD of 0.52 (95% CI: 0.33–0.71, *p* < 0.00001; *I*^2^ = 51%); similar effects were exerted on appendicular lean mass (SMD 0.64, 95% CI: 0.34–0.93, *p* < 0.0001; *I*^2^ = 0%; LoE, strong).

Two RCTs reported changes in fat mass after PS plus exercise training [48,58]. The results of the meta-analysis revealed a conflicting evidence of the effects of PS plus exercise training on fat mass (Figure 3 and Figure S9).

A subgroup analysis of exercise training type revealed that PS plus RET exhibited significant effects on lean body mass and appendicular skeletal muscle mass gains with an SMD of 0.61 (95% CI: 0.35–0.88, *p* < 0.00001; *I*^2^ = 34%) and 0.75 (95% CI: 0.32–1.18, *p* = 0.0006; *I*^2^ = 0%), respectively, whereas PS plus MET did not (Table 4).

The results of subgroup analyses (Table 4) showed that all factors generally had no effect on subgroup heterogeneity for lean body mass, appendicular lean mass, and fat mass (all *p* > 0.05), except that significant subgroup differences in heterogeneity were observed between different exercise types (*p* = 0.04, *I*^2^ = 75.3%) and supplementation doses (*p* = 0.04, *I*^2^ = 76.7%) for lean body mass. High-dose supplementations appeared to exert significant effects on lean body mass with an SMD of 0.71 (*p* < 0.00001; LoE, strong), whereas PS plus MET and low-dose supplementation did not.

### 3.9. Effects on Leg Strength and Physical Mobility Outcome

The effect of PS plus exercise training on changes in leg strength in frail older individuals was determined by measuring the quadriceps muscle power by two RCTs [43,51], leg press one-repetition maximum (1-RM) strength by five RCTs [48,49,57,58,61], maximum isometric knee strength by four RCTs [45,52,54,59], and isokinetic knee strength by two RCTs (Table 1) [55,56]. The combined analysis derived from the 13 RCTs (15 comparisons) showed a significant effect of PS plus exercise training on changes in leg strength, with an SMD of 0.37 (95% CI: 0.23–0.51; *p* < 0.00001; *I*^2^ = 37%; LoE, strong) in the overall follow-up duration (Figure 3 and Figure S10).

The treatment effect of PS plus exercise training on physical function was assessed using several mobility tests, namely chair rise time by eight RCTs [41,43,45,48,50,56,58,59], timed up-and-go by four RCTs [52,54,55,59], and short physical performance battery by three RCTs [46,48,58], as well as ADL by five RCTs [42,45,52,53,59]. Forest plots for all physical function measures demonstrated a statistically significant effect in favor of PS plus exercise training (all *p* < 0.05; LoE, strong) with exception of the ADLs (Figure 3 and Figures S11–S14).

A subgroup analysis of exercise training type revealed that both PS plus RET and PS plus MET exhibited significant effects on leg strength with an SMD of 0.37 (95% CI: 0.04–0.07, *p* = 0.03; *I*^2^ = 57%) and 0.37 (95% CI: 0.17–0.58, *p* = 0.0004; *I*^2^ = 24%), respectively; similar results were observed in chair stand and timed up-and-go performances.

The results of subgroup analyses (Table 5) showed that all factors generally exerted no effects on subgroup heterogeneity for leg strength and mobility (all *p* > 0.05), except that significant subgroup differences in heterogeneity between different participant types (*p* = 0.01, *I*^2^ = 83.2%) and population areas (*p* = 0.002, *I*^2^ = 84.5%) were observed for leg strength. The frail institutionalized residents appeared to respond to PS plus exercise training with a greater SMD in leg strength of 0.57 (95% CI: 0.36–0.78, *p* < 0.00001) compared with community-dwelling peers (SMD: 0.23, 95% CI: 0.05–0.41, *p* = 0.01).

### 3.10. Publication Bias

**Figure 4:**
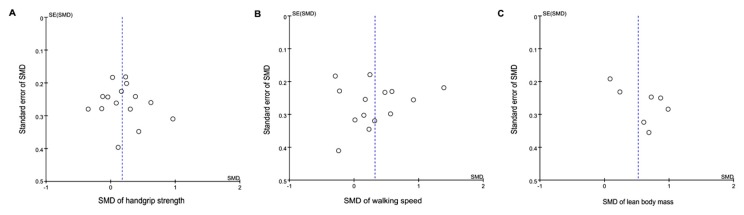
Funnel plots of the intervention effects for (**A**) handgrip strength, (**B**) walking speed, and (**C**) lean body mass. Each circle represents an independent comparison, with the *x*-axis representing standard mean difference (SMD) over control comparisons and the y-axis showing the standard error (SE) of SMD. The vertical dotted line indicates the mean value of the SMDs.

The visual inspection of a funnel plot of increase in handgrip strength, walking speed, and lean body mass did not reveal substantial asymmetry (Figure 4). Egger’s linear regression test for handgrip strength also indicated no evidence of obvious reporting bias among the RCTs (t = 0.06, *p* = 0.95); similar results were obtained for walking speed (t = −0.07, *p* = 0.91) and lean body mass (t = −0.76, *p* = 0.48).

## 4. Discussion

Compared with previous systematic reviews on the efficacy of PS plus exercise training [15,17,18,19,20], this study focused on the frail older individuals and investigated pooled results of frailty indices based on the Fried criteria [24]. The major findings of the present study are summarized in Table 6 which showed moderate to strong evidence that regardless of the follow-up duration, PS plus RET or MET exerted overall significant effects on frailty indices as well as body composition (lean body mass and appendicular lean mass), leg strength, and physical mobility in community-dwelling or institutionalized frail older individuals.

In this meta-analysis, the results of subgroup analyses based on the control type showed that PS plus exercise training exerted significant greater effects on frailty indices (including whole body mass, handgrip strength, and exhaustion), lean body mass, and leg strength compared with exercise training alone. These results coincide with the findings of our previous studies, indicating that an additional PS augments lean body mass and strength gain during RET in older adults [20,62]. The results of this study further indicated that an additional PS may have effects on improvement of frailty status and body composition among frail older individuals undergoing exercise training sessions, regardless of the supplementation dose and exercise training type. In agreement with previous reviews [63,64] and following the recommendations from the European Society for Clinical Nutrition and Metabolism Expert Group [65], the results of the current meta-analysis supported the urgent necessity for prefrail or frail older adults to incorporate protein-based nutrition intervention plus exercise training to prevent functional decline, especially for institutionalized residents who are at a high risk of insufficient protein intake and physical inactivity [66,67,68,69].

PS in combination with resistance-type exercise training has been identified as an efficient intervention for lean mass and strength gain in the older population [20,21,65,70,71,72]. However, an RET intensity as high as 80–95% 1-RM has been recommended to induce maximal muscle hypertrophy or muscle fiber adaptation [73,74], which is not allowed for most frail older adults, especially for the institutionalized residents who are more dependent and usually have lower exercise adherence rate due to cardiopulmonary dysfunction or physical limitations [75]. Therefore, physical activity exercises combining RET with aerobic exercises, balance training, and functional mobility training (i.e., MET) are recommended for older adults to improve physical function and prevent falls [64,76,77]. Most of the included RCTs in this meta-analysis employed RET with a moderate–to-high intensity of 50–80% 1-RM [48,49,50,51,56,58,61], whereas MET was mostly performed with a light-to-moderate intensity for frail older participants [26,41,42,45,46,52,54,59]; the results showed that PS plus MET as well as PS plus RET had significant effects on whole body mass, walking speed, and leg strength, which indicated that frail older adults responded favorably to PS plus MET in reversing or preventing frail status. In addition, results of subgroup analyses in the present study showed no difference in all outcome measures between two participant types, except that the nursing-home residents exhibited a greater effect on leg-strength gain compared to the frail community-dwellers; this further indicated that the nursing-home residents may as well have intervention outcomes in responding to PS plus MET or RET as the frail community-dwellers did. However, we also observed that PS plus RET yielded greater SMDs for lean body mass and appendicular lean mass compared with PS plus MET, which was also supported by previous results regarding the intensity of RET for muscle hypertrophy [74] and further indicated that PS plus RET may have greater efficiency in muscle mass gain than PS plus MET did. Taken together, we conclude that PS plus MET may exert significant effects on frailty indices, whereas PS plus RET further improves muscle mass loss in frail older individuals.

Regarding for the amount of PS, an adequate protein-enriched diet or sufficient PS enhances myofibrillar protein synthesis [18], despite the lower fractional synthetic rate in healthy elderly individuals compared with young peers [78,79,80]. Based on the dose-dependent responses of PS responding to yield an increased anabolic resistance of age [38,81], a sufficient PS up to 40 g or 1.6 g/kg/d after RET has been believed to augment the effects of resistance training on muscle mass gain in older adults [19,21]. However, the need for PS in sarcopenic or frail older individuals to overcome a blunted anabolic response to diet protein remains uncertain because of the potential confounding factors involved, such as deficit energy metabolism and mitochondrial dysfunction, which may influence the PS efficacy [82,83]. In addition, the previous studies regarding PS for the older individuals demonstrated conflicted results; some authors identified the effects of PS on muscle mass accretion and strength gain during exercise training in sarcopenic or frail individuals [54,58,84,85], whereas others concluded that PS provides no additional benefit in trained community-dwelling or institutionalized residents [44,48,49,56,86]. In this study, a cut off value of 30 g/day (or g/session) was used for subgroup analyses to identify the influence of supplementation dose in intervention effects; the results showed that supplementation dose had no effect on subgroup heterogeneity for all outcome measures, except whole body mass and lean body mass (Table 6); it indicated that the amount of PS daily or after exercise training may play an important role in muscle mass gain rather than strength gain or physical function restoration, especially for the frail older individuals.

Previous systematic reviews have demonstrated nonsignificant effects on changes in body weight [21,71,87], muscle mass [19,87], muscle strength [19], and physical mobility [20] in response to PS plus RET among older adults who mostly were healthy or nonfrail. The difference between the results of previous reviews and the findings in the present meta-analysis may be attributed to different populations, which further confirm the conclusion of previous authors that frail individuals may have greater benefits in body composition and physical performance in response to PS plus exercise training than their healthy peers [71,88]. Therefore, targeting the frailty indices in response to PS plus exercise training may hold more promise in the preservation of independence as well as the prevention of progress to frailty in the older population.

Several limitations to our findings should be elucidated. First, based on the varying PS protocols (protein source, supplied amounts, and timing of ingestion) and exercise regimes (training duration and training volume), it was difficult to endorse a definite conclusion regarding the effect of a specific type of PS or exercise training on frailty indices, lean body mass gain, or strength gain. Second, some of the included RCTs had a small sample size [43,48,59], and the results of the studies that reflected no significant intervention effect on primary or secondary outcomes may contribute negatively to the overall effect size. Third, some of the included RCTs had participants with impaired cognition [26,41,42,53]. Demented older individuals are more dependent and usually have lower adherence rates of exercise or nutrient interventions which may influence the intervention effects. Fourth, we included RCTs with an attendance rate (or compliance) lower than 80% responding to exercise training [41,43,51,56,57] and protein supplementations [43]; in addition, 6 and 8 of 22 RCTs did not reported compliance to exercise and PS interventions. Based on that participants with higher attendance or motivation at the training sessions have better performance in mobility [50,56], including the RCTs with low attendance could have impacts on the present results. Fifth, inadequate statistical power for subgroup analyses was noted. Several subgroups (such as participant types for lean body mass) included a small number of RCTs less than six, which may not have adequate power for detecting a difference among subgroups [89,90]; the results of such subgroup analyses should be cautiously interpreted. Finally, there may have been sex differences for changes in body composition in response to PS plus exercise training [20,91,92,93]. Because few RCTs with a sex-specific methodological design were available, we could not perform subgroup analyses based on sex for identifying sex influence on treatment effects. However, one included RCT reported the total improvement rates of frailty indices for male and female participants separately [55], and treatment success rates for the diminishing frailty status were similar between frail older men (OR: 2.00, 95% CI: 0.09–44.35) and women (OR: 2.86, 95% CI: 0.41–20.14); and one RCT reported gender differences with higher values for elder men in muscle strength and walking speed [56]. Additional sex-specific studies on frail population are warranted to confirm the evidence of such sex influence on the treatment effects of PS plus exercise training.

## 5. Conclusions

This systematic review provided strong evidence that PS combined with exercise training is effective for improving frailty indices, promoting gains in muscle mass and strength, and enhancing performance in physical mobility in frail older adults compared with placebo, PS-alone, and exercise-alone controls. In addition, PS plus MET exerted relevant effects on diminishing frail status, whereas PS plus RET exerted additional effects on muscle mass gain. Therefore, we concluded that PS in combination with RET or MET may exert additional effects to prevent (or offset) muscle loss and functional decline, especially for older individuals who are frail community dwellers or institutionalized residents. The study results provide insights into effective nutrition and exercise intervention strategies and interdisciplinary approach practices to counteract muscle loss and functional decline in this population. This is relevant for those working in elder care and rehabilitation such as in clinical, hospitalized, institutionalized, and community settings. Because of the limitations of our current study, additional studies with larger samples and the identification of specific supplementation protocols are necessary.

## Figures and Tables

**Table 1:** Summary of study characteristics of included studies.

Study (Author, Year, Ref.)	Country (Area)	Groups *^1^*	Age (y) *^2^*	Sex (F/M)	*N*	Design	BMI (kg/m^2^) *^2^*	Patient Type	Body Composition Assessment Method	Exercise Intervention		Protein Supplement		Measured Time Point	Outcome Results
										Type, Compliance (%, EG/CG)	Frequency × Duration	Type, Compliance (%, EG/CG) *^10^*	Intake Amount (g/day or g/session)		
Beck	Denmark	EG: PS + ET	86.0 ± 8.4	41/14	55	RCT	20.7 ± 4.0	Nursing-	NA	MET	2 day/week × 11 weeks	Proteins	18.0 g/session	Baseline	↑ CRT*^7^*; HG*^8^*
2016 [41]	(Europe)	CG: Control*^3^*	87.3 ± 7.6	30/10	40		21.1 ± 3.3	home residents		71/NA	(22 sessions)	100/NA		Posttest: 11 weeks	
Beck	Denmark	EG: PS + ET	87 (84–90)	42/20	62	RCT	23.4 (21.8–24.8)	Nursing-	DXA	MET	2 day/week × 11 weeks	Milk protein	7.0 g/day	Baseline	↑ TUG*^7^*; ↑ HG*^7^*
2008 [26]	(Europe)	CG: Control*^3^*	86 (84–87)	46/13	59	SB	23.4 (21.3–25.2)	home residents		100/100	(22 sessions)	100/100	3.0 g/session	Posttest: 11 weeks	↑ BBS*^7^*; ↑ CRT*^7^*
2010 [42]														Follow-up: 27 weeks	ADL*^8^*; ↑ MDS-CPS*^7^*
Bonnefoy	France	EG: ET + PS	83.5 ± 1.2*^9^*	50/7*^9^*	57*^9^*	RCT	27.2 ± 0.9*^9^*	Frail older	DLW	MET	3 day/week × 36 weeks	Proteins	30.0 g/day	Baseline	FFM*^8^*; GS*^8^*; SC*^8^*
2003 [43]	(Europe)	CG 1: ET + PLA-S				SB		Individuals	method	63-70*^9^*	(108 sessions)	61/54		Midtest: 12 weeks	↑ Leg strength*^7^*
		CG 2: PLA-S												Posttest: 36 weeks	
Carlsson	Sweden	EG: PS + ET	84.4 ± 6.3	33/9	42	RCT	25.1 ± 4.6	Frail older	BIA	MET	2–3 day/week × 13 weeks	Milk protein	7.4 g /session	Baseline	ICW*^8^*; FM*^8^*; BBS*^8^*
2011 [44]	(Europe)	CG 1: ET + PLA-S	85.3 ± 5.5	28/13	41	DB	25.2 ± 4.4	individuals		79/72	(29 sessions)	84/79		Posttest: 12 weeks	
		CG 2: PS	82.7 ± 6.4	34/13	47		24.9 ± 4.5							Follow-up: 24 weeks	
		CG 3: PLA-S	85.4 ± 7.2	36/11	47		24.6 ± 4.9								
Chin A	Netherlands	EG: PS + ET	78.9 ± 6.0	31/11	42	RCT	25.0 ± 2.5	Frail older	NA	MET	2 day/week × 17 weeks	Proteins	20.0 g/day	Baseline	↑ GS*^7^*; ↑ TUG*^7^*
Paw	(Europe)	CG 1: ET	76.2 ± 4.5	28/11	39	DB	24.4 ± 2.9	individuals		90 (47–100)	(34 sessions)	85/32		Posttest: 17 weeks	
2001 [45]		CG 2: PS	79.2 ± 4.8	28/11	39		24.5 ± 2.4								
		CG 3: PLA-S	78.6 ± 6.6	20/17	37		24.1 ± 3.1								
Corcoran	America	EG: PS + ET	82.3 ± 7.6	56/11	67	RCT	28.2 ± 4.3	Frail facility	NA	MET	3 day/week × 24 weeks	Milk protein	20.0 g /session	Baseline	↑ PA*^6,8^*; ↑ HG*^6,8^*
2017 [46]	(Americas)	CG: Control*^3^*	81.2 ± 8.5	45/9	54	SB	28.5 ± 4.7	residents		81/68	(72 sessions)	87/NA		Midtest: 12 weeks	SPPB*^8^*; GS*^8^*
														Posttest: 24 weeks	
de Jone	Netherlands	EG: PS + ET	78.8 ± 6.1	28/11	39	RCT, SB	24.9 ± 2.5	Frail older	NA	MET	2 day/week × 17 weeks	Proteins	20.0 g/day	Baseline	WBM*^8^*
1999 [47]	(Europe)	CG 1: ET	76.5 ± 4.5	25/10	35		24.3 ± 3.1	individuals		NR	(34 sessions)	NR		Posttest: 17 weeks	
		CG 2: PS	78.9 ± 4.8	26/11	37		24.3 ± 2.3								
		CG 3: PLA-S	78.7 ± 6.8	23/11	34		24.1 ± 3.2								
Dirks	Netherlands	EG: PS + ET	76.0 ± 8.2	11/6	17	RCT	29.5 ± 4.9	Frail older	DXA	RET	2 day/week × 24 weeks	Milk protein	30.0 g /session	Baseline	↑ LBM*^6,^^7^*; ↑ ALM*^6,^^7^*; ↑ FM*^6,^^7^*
2017 [48]	(Europe)	CG: ET + PLA-S	77.0 ± 8.2	11/6	17	DB	28.6 ± 3.7	individuals		84±2*^9^*	(48 sessions)	NR		Midtest: 12 weeks	↓ CRT*^5,^^6^*; ↑ LP 1-RM*^5,^^6^*
														Posttest: 24 weeks	↑ Muscle fiber CSA*^6^*
Fiatarone	America	EG: PS + ET	87.2 ± 6.0	16/9	25	RCT	24.5 ± 4.0	Nursing-	WBP	RET	3 day/week × 10 weeks	Soy protein	40.8 g/day	Baseline	WBP*^8^*; ↑ GS*^6,^^7^*; ↑ SC*^6,^^7^*
1994 [49]	(Americas)	CG 1: ET + PLA-S	86.2 ± 5.0	16/9	25	DB	24.9 ± 3.5	home residents	method	97/100	(30 sessions)	99/100		Posttest: 10 weeks	↑ leg strength*^6^*^,*7*^
		CG 2: PS	85.7 ± 5.8	17/7	24		25.4 ± 4.9								Quadriceps CSA*^8^*
		CG 3: PLA-S	89.2 ± 4.1	14/12	26		25.8 ± 5.1								↑ LP 1-RM*^6,^^7^*; ↑ PA*^6,^^7^*
Franzke	Australia	EG: PS + ET	82.5 ± 7.5	84/13*^9^*	29	RCT	NR	Institutionalized	NA	RET	2 day/week × 24 weeks	Whey protein,	20.7 g/day	Baseline	↑ HG*^7^*; ↓ CRT*^5,6^*^,*7*^
2015 [50]	(Oceania)	CG 1: ET	82.8 ± 5.7		35			older		NR	(48 sessions)	Leucine, EAA,	41.4 g /session	Midtest: 12 weeks	↑ 6MWD*^5^*^,^*^6^*^,*7*^
		CG 2: Control*^3^*	83.5 ± 5.4		33			individuals				NR		Posttest: 24 weeks	
Hofmann	Austria	EG: PS + ET	84 (65–92)	9/19	28	RCT	28.7 (22.9, 50.0)	Institutionalized	BIA	RET	2 day/week × 24 weeks	Leucine, EAA	20.7 g/day	Baseline	SMM*^8^*; HG*^8^*
2016 [51]	(Europe)	CG 1: ET	83 (72–92)	12/11	33		29.0 (22.7, 40.2)	older women		71±26.5*^9^*	(48 sessions)	NR	41.4 g /session	Midtest: 12 weeks	↑ Leg MQ*^5,6^*^,*7*^
		CG 2: Control*^3^*	85 (69–92)	10/20	30		29.7 (18.1, 36.9)							Posttest: 24 weeks	
Ikeda	Japan	Tr 1 EG: PS + ET	78.4 ± 7.8	9/18	27	RCT, DB	23.6 ± 3.4	Frail older	NA	MET	2 day/week × 12 weeks	EAA	6.0 g/session	Baseline	↑ LP 1-RM*^6^*; HG*^8^*; TUG*^8^*
2016 [52]	(Asian)	Tr 1 CG: ET + PLA-S	80.4 ± 8.9	10/15	25	Crossover	21.9 ± 3.4	individuals		97.7/97.2	(24 sessions)	100/100		Posttest: 12 weeks	Leg strength*^8^*; FRT*^6^*^,*7*^
		Tr 2 EG: PS + ET	80.4 ± 8.9	10/15	25		21.6 ± 3.8				2 day/week × 12 weeks			Baseline	↑ LP 1-RM*^6^*; HG*^8^*; TUG*^8^*
		Tr 2 CG: ET + PLA-S	78.4 ± 7.8	9/18	27		23.5 ± 3.5				(24 sessions)			Posttest: 12 weeks	Quad strength*^8^*; FRT*^6^*
Imaoka	Japan	EG: PS + ET	87.6 ± 6.5	18/5	23	RCT	20.4 ± 3.7	Institutionalized	BIA	MET	2 day/week × 12 weeks	Proteins	4.1 g/day	Baseline	SMI*^8^*; FIM*^8^*
2016 [53]	(Asian)	CG 1: ET	82.6 ± 9.1	16/6	22		20.5 ± 3.2	frail older		NR	(24 sessions)	NR		Posttest: 12 weeks	↓ Incidence of falls*^7^*
		CG 2: PS	84.6 ± 7.7	20/3	23		20.4 ± 3.3	individuals						Follow-up: 26 weeks	
		CG 3: Control*^3^*	82.5 ± 10.9	15/8	23		20.6 ± 3.1								
Kim	Japan	EG: PS + ET	81.0 ± 2.6	33/0	33	RCT	21.1 ± 3.6*^4^*	Frail older	DXA	MET	2 day/week × 12 weeks	Milk protein	22.0 g/day	Baseline	ALM*^8^*; LLM*^8^*; GH*^8^*
2015 [54]	(Asian)	CG 1: ET + PLA-S	81.1 ± 2.8	33/0	33	DB	22.2 ± 4.3*^4^*	women		NR	(24 sessions)	NR	22.0 g /session	Posttest: 12 weeks	↑ GS*^7^*; ↑ TUG*^7^*
		CG 2: PS	81.0 ± 2.8	32/0	32		22.1 ± 4.2*^4^*							Follow-up: 28 weeks	Leg strength*^8^*
		CG 3: PLA-S	80.3 ± 3.3	33/0	33		22.9 ± 4.3*^4^*								↓ Frailty score*^5,6,7^*
Niccoli	Canada	EG: PS + ET	81.8 ± 1.7	15/7	22	RCT	24.2 ± 5.2	Hospitalized	NA	MET	7 day/week × 4 wk*^1^*^0^	Whey protein	24.0 g/day	Baseline	↑ GS*^5^*^,^*^6^*^,*7*^; ↑ HG*^6^*^,*7*^
2017 [55]	(Americas)	CG: ET + PLA-S	80.3 ± 1.6	17/8	25	DB	26.4 ± 6.6	frail older individuals		NR	(28 sessions)	NR		Posttest: IPDC*^11^*	↓ TUG*^5^*^,*6*^; ↑ Leg strength*^6,7^*
Oesen	Austria	EG: PS + ET	81.8 ± 6.9	37/4	41	RCT	29.8 ± 6.1	Institutionalized	NA	RET	2 day/week	Leucine, EAA	20.7 g/day	Baseline	↑ Hand lifting*^5,6^*^,*7*^; ↓ CRT*^5,6^*^,*7*^
2015 [56]	(Europe)	CG 1: ET	83.0 ± 5.5	31/5	36		28.9 ± 3.7	older		71±26.5*^9^*	× 24 weeks	NR	41.4 g /session	Midtest: 12 weeks	↑ Leg power*^5,6^*^,*7*^; ↑ GS*^5^*^,^*^6^*^,*7*^
		CG 2: Control*^3^*	83.4 ± 5.6	35/5	40		28.9 ± 5.0	individuals			(48 sessions)			Posttest: 24 weeks	↑ 6MWD*^5^*^,^*^6^*^,*7*^; ↑ PA*^5^*^,^*^6,^^7^*; FRT*^8^*
Rosendahl	Sweden	EG: PS + ET	85.0 ± 6.7	36/10	46	RCT	24.9 ± 4.6	Institutionalized	NA	MET	2−3 day/week	Milk protein	7.4 g /session	Baseline	↑ Leg power*^5,6^*^,*7*^
2006 [57]	(Europe)	CG 1: ET + PLA-S	85.6 ± 5.5	31/14	45	DB	24.8 ± 4.4	frail older		72/70	× 13 weeks	82/78		Posttest: 13 weeks	↑ GS*^5^*^,^*^6^*^,*7*^; CRT*^6^*^,*7*^
		CG 2: PS	82.9 ± 6.4	35/15	50		24.9 ± 4.4	individuals			(29 sessions)			Follow-up: 24 weeks	↑ BBS*^5^*^,^*^6^*^,*7*^
		CG 3: PLA-S	85.6 ± 7.0	37/13	50		24.5 ± 4.9								
Tieland	Netherlands	EG: PS + ET	78.0 ± 9.0	21/10	31	RCT	28.7 ± 4.5	Frail older	DXA	RET	2 day/week × 24 wk	Milk protein	30.0 g/day	Baseline	↑ LBM*^6,^^7^*; ↑ ALM*^6,^^7^*
2012 [58]	(Europe)	CG: ET + PLA-S	79.0 ± 6.0	20/11	31	DB	28.2 ± 4.6	individuals		≥98*^9^*	(48 sessions)	≥98*^9^*		Midtest: 12 weeks	↑ FM*^6,^^7^*; ↑ LP 1-RM*^5,^^6^*
														Posttest: 24 weeks	↓ CRT*^5,^^6^*
Trabal	Spain	EG: PS + ET	85.0 ± 8.0	16/8*^9^*	12	RCT	26.6 ± 4.4	Institutionalized	NR	MET	4 day/week × 12 weeks	Leucine	10.0 g/day	Baseline	↑ Leg strength*^7^*; ↓ TUG*^7^*
2015 [59]	(Europe)	CG: ET + PLA-S	84.0 ± 4.0		12	DB	26.0 ± 5.2	older		98±3/96±8	(48 sessions)	80±14/95±5		Midtest: 4 weeks	↑ Arm girth*^7^*; SLS*^8^*
								individuals						Posttest: 12 weeks	GS*^8^*; CRT*^8^*; SF-36 PF*^8^*
Yamada	Japan	EG: PS + ET	78.1 ± 5.7	19/12	31	RCT	22.1 ± 3.6	Frail older	BIA	Weighted	7 day/week × 24 weeks	Protein	10.0 g/day	Baseline	↑ SMI*^7^*
2015 [60]*^12^*	(Asian)	CG 1: ET	75.7 ± 5.8	8/7	15		22.6 ± 3.1	individuals		walking	(168 sessions)	(BCAA)		Posttest: 24 weeks	
		CG 2: Control*^3^*	76.4 ± 6.2	15/10	25		23.2 ± 3.2			NR		80 (67−92)			
Zak	Poland	EG 1: PS + RET	78.1 ± 7.6	16/3	19	RCT	24.7 ± 0.8	Frail older	NA	RET	5 day/week × 7 weeks	Proteins	32.0 g/day	Baseline	↑ 6MWD*^5^*^,^*^6^*
2009 [61]	(Europe)	CG 1: RET + PLA-S	79.2 ± 9.2	17/4	21	DB	24.3 ± 0.6	individuals		100/100	(35 sessions)	100/100		Posttest: 7 weeks	↑ Tinetti-TS*^5^*
		EG 2: PS + SE	78.3 ± 6.8	14/5	19		25.2 ± 0.7			MET					↑ Leg strength*^5,6^*
		CG 2: SE + PLA-S	81.1 ± 6.4	17/4	21		25.2 ± 0.6			100/100					

*^1^* Groups with PS + ET are presented as EG, otherwise is presented as CG; *^2^* Values are presented as mean and SD (or range); *^3^* A group without any nutrient supplement and exercise training; *^4^* Data were estimated. *^5^* Significant within-group difference for control compared with baseline. *^6^* Significant within-group difference for PS + ET compared with baseline. *^7^* Significant between-group difference for PS + ET compared with control. *^8^* Nonsignificant between-group difference for PS + ET compared with control. *^9^* Values of all samples. *^1^*^0^ Values denote the compliance of protein and placebo supplement (%) in EG and CG, respectively. *^11^* Mean length of hospital stay. *^12^* Only frail participants’ data were extracted. 6MWD, 6-min walk-for-distance; ADL, activity of daily living; ALM, appendicular lean mass; BBS, Berg’s balance scale; BCAA, branched chain amino acids; BIA, bioelectrical impedance analysis; BMI, body mass index; CG, control group; CRT, chair rise time; CSA, cross-sectional area; CT, cognition training; DB, double blind; DLW, doubly labeled water; DXA, dual-energy X-ray absorptiometry; EAA, essential amino acids; EG, experimental group; ET, exercise training; FIM, functional independence measure; FFM, fat-free mass; FM, fat mass; FRT, functional reach test; GS, gait speed; HG, handgrip strength; IADL, Instrumental Activities of Daily Living; ICW, intracellular water; IPDC, inpatient discharge; LBM, lean body mass; LLM, leg lean mass; LP 1-RM, leg press one repetition maximum; MDS-CPS, Resident Assessment Instrument, Minimum Data Set-subscale on cognitive performance levels; MET, multicomponent exercise training; MQ, muscle quality; NA, not applicable; NR, not reported; PA, physical activity; PASE, Physical Activity Scale for the Elderly; PLA-S, placebo supplement; PS, protein supplementation; RCT, randomized controlled trial; Ref = reference number; RET, resistance exercise training; SB, single blind; SC, stair climbing; SE, standard exercise; SF-36 PF, Short-Form 36-Item Health Survey (physical function subscore); SMI, skeletal muscle mass index; SMM, skeletal muscle mass; SLS, single leg stance; SPPB, short physical performance battery; Tinetti-TS, Tinetti total score; Tr, treatment session; TUG, timed up-and-go test; WBM, whole body mass; WBP, whole body potassium; ↑, significant increase; ↓, significant decrease.

**Table 2:** Summary of methodological quality based on the PEDro classification scale ^a^.

Study Author (Year) (Reference Number)	Overall ^b^	Eligibility Criteria ^c^	1	2	3	4	5	6	7	8	9	10
Beck 2016 [41]	6/10	*X*	*X*		*X*				*X*	*X*	*X*	*X*
Beck 2008 [26]	6/10	*X*	*X*		*X*			*X*		*X*	*X*	*X*
Beck 2010 [42]	6/10	*X*	*X*		*X*			*X*		*X*	*X*	*X*
Bonnefoy 2003 [43]	7/10	*X*	*X*		*X*	*X*			*X*	*X*	*X*	*X*
Carlsson 2011 [44]	9/10	*X*	*X*	*X*	*X*	*X*	*X*	*X*	*X*		*X*	*X*
Chin A Paw 2001 [45]	9/10	*X*	*X*	*X*	*X*	*X*		*X*	*X*	*X*	*X*	*X*
Corcoran 2017 [46]	6/10	*X*	*X*		*X*			*X*		*X*	*X*	*X*
de Jone 1999 [47]	4/10		*X*		*X*						*X*	*X*
Dirks 2017 [48]	7/10	*X*	*X*		*X*	*X*			*X*	*X*	*X*	*X*
Fiatarone 1994 [49]	8/10	*X*	*X*		*X*	*X*		*X*	*X*	*X*	*X*	*X*
Franzke 2015 [50]	5/10	*X*	*X*		*X*			*X*			*X*	*X*
Hofmann 2016 [51]	5/10		*X*		*X*				*X*		*X*	*X*
Ikeda 2016 [52]	7/10	*X*	*X*		*X*	*X*	*X*			*X*	*X*	*X*
Imaoka 2016 [53]	6/10	*X*	*X*	*X*	*X*				*X*		*X*	*X*
Kim 2015 [54]	9/10	*X*	*X*	*X*	*X*	*X*		*X*	*X*	*X*	*X*	*X*
Niccoli 2017 [55]	8/10	*X*	*X*		*X*	*X*	*X*		*X*	*X*	*X*	*X*
Oesen 2015 [56]	5/10	*X*	*X*		*X*					*X*	*X*	*X*
Rosendahl 2006 [57]	8/10	*X*	*X*	*X*	*X*			*X*	*X*	*X*	*X*	*X*
Tieland 2012 [58]	7/10	*X*	*X*		*X*	*X*	*X*		*X*		*X*	*X*
Trabal 2015 [59]	5/10	*X*	*X*		*X*	*X*	*X*				*X*	*X*
Yamada 2015 [60]	6/10	*X*	*X*		*X*				*X*	*X*	*X*	*X*
Zak 2009 [61]	8/10	*X*	*X*		*X*	*X*	*X*	*X*	*X*		*X*	*X*
Summary ^#^		20	22	5	22	11	6	10	14	14	22	22

^a^ PEDro = Physiotherapy evidence database. Guidelines of the PEDro scale is available at the PEDro database (https://www.pedro.org.au/english/downloads/pedro-scale/). ^b^ Points of methodological quality are denoted as “*X*” for fulfilled criteria. ^c^ Not used to calculate the total score. Score was determined by a third assessor. ^#^This was calculated as the number of studies satisfied. PEDro classification scale: 1 = random allocation, 2 = concealed allocation, 3 = similarity at the baseline, 4 = subject blinding, 5 = therapist blinding, 6 = assessor blinding, 7 = more than 85% follow-up for at least one key outcome, 8 = intention-to-treat analysis, 9 = between-group statistical comparison for at least one key outcome, 10 = point and variability measures for at least one key outcome. Methodological quality: high, ≥7 points; medium, 4–6 points; low, ≤3 points.

**Table 3:** Summary of overall effects and subgroup analysis results for frailty indices.

Subgroup	Global Frailty Score ^a^					Whole Body Mass					Handgrip Strength				
	Comparison, *n* (LoE) ^b^	SMD	(95%CI)	*p* Value	*I*^2^ (%)	Comparison, *n* (LoE)^b^	SMD	(95%CI)	*p* Value	*I*^2^ (%)	Comparison, *n* (LoE)^b^	SMD	(95%CI)	*p* Value	*I*^2^ (%)
Overall	1 (M)	0.62	(0.21, 1.03)	0.003	NA	11 (S)	0.38	(0.23, 0.52) ^†^	<0.00001	37	15 (S)	0.17	(0.05, 0.30) ^†^	0.006	26
MQ level (PEDro score)															
≥7/10	1 (M)	0.62	(0.21, 1.03)	0.003	NA	6 (M)	0.58	(0.24, 0.92) ^‡^	0.0009	65	7 (S)	0.31	(0.11, 0.50) ^†^	0.002	50
<7/10	0					5 (M)	0.24	(0.02, 0.46) ^†^	0.03	0	8 (C)	0.08	(0.08, 0.24) ^†^	n.s.	0
Subgroup difference				NA	NA				n.s.	62.3				n.s.	54
Participant type															
Community dweller	1 (M)	0.62	(0.21, 1.03)	0.003	NA	6 (M)	0.58	(0.25, 0.91) ^‡^	0.0006	65	7 (S)	0.18	(0.01, 0.36) ^†^	0.04	24
Institutionalized resident	0					5 (C)	0.22	(−0.02, 0.45) ^†^	n.s.	0	8 (C)	0.17	(−0.01, 0.34) ^†^	n.s.	37
Subgroup difference				NA	NA				n.s.	66.5				n.s.	0
Population area															
Americas	0					1 (C)	0.41	(−0.06, 0.88)	n.s.	NA	2 (C)	0.46	(−0.45, 1.37) ^‡^	n.s.	85
Asia	1 (M)	0.62	(0.21, 1.03)	0.003	NA	1 (M)	0.66	(0.26, 1.06)	0.001	NA	4 (C)	0.05	(−0.19, 0.29) ^†^	n.s.	31
Europe	0					9 (S)	0.33	(0.16, 0.49) ^†^	0.0001	41	8 (S)	0.24	(0.06, 0.42) ^†^	0.007	0
Oceania	0					0					1 (C)	−0.05	(−0.52, 0.43)	n.s.	NA
Subgroup difference				NA	NA				n.s.	0				n.s.	0
Control group type															
PLA-S or nonexercise	1 (M)	1.08	(0.56, 1.60)	<0.0001	NA	7 (S)	0.43	(0.22, 0.64) ^†^	<0.0001	38	9 (S)	0.09	(−0.09, 0.26) ^†^	n.s.	15
Exercise	1 (C)	0.34	(−0.14, 0.83)	n.s.	NA	8 (S)	0.32	(0.14, 0.50) ^†^	0.0005	26	11 (S)	0.28	(0.12, 0.43) ^†^	0.0007	29
PS	1 (M)	0.63	(0.13, 1.13)	0.01	NA	4 (S)	0.37	(0.11, 0.63) ^†^	0.006	56	2 (L)	0.16	(−0.22, 0.54) ^†^	n.s.	74
Subgroup difference				n.s.	52.1				n.s.	0				n.s.	4.7
Supplementation dose															
≥30 g/day (g/session)						4 (S)	0.65	(0.36, 0.92 )^†^	<0.00001	35	5 (S)	0.28	(0.04, 0.51) ^†^	0.02	10
<30 g/day (g/session)	1 (M)	0.62	(0.21, 1.03)	0.003	NA	7 (S)	0.26	(0.07, 0.46) ^†^	0.001	9	10 (C)	0.13	(−0.01, 0.28) ^†^	n.s.	34
Subgroup difference				NA	NA				0.03	78.2				n.s.	0
Exercise type															
RET	0					4 (M)	0.44	(0.03, 0.84) ^‡^	0.04	63	6 (S)	0.26	(0.07, 0.46) ^†^	0.008	0
MET	1 (M)	0.62	(0.21, 1.03)	0.003	NA	7 (S)	0.38	(0.19, 0.56) ^†^	<0.0001	21	9 (C)	0.11	(−0.05, 0.28) ^†^	n.s.	39
Subgroup difference				NA	NA				n.s.	0				n.s.	0
Intervention duration															
<12 weeks	0					5 (C)	0.19	(−0.04, 0.42) ^†^	n.s.	0	4 (C)	0.29	(−0.14, 0.72) ^‡^	n.s.	59
12–24 weeks	1 (M)	0.62	(0.21, 1.03)	0.003	NA	7 (S)	0.38	(0.20, 0.57) ^†^	<0.0001	19	11 (C)	0.09	(−0.05, 0.23) ^†^	n.s.	0
≥24 weeks	0					4 (C)	0.43	(−0.02, 0.87) ^‡^	0.04	64	6 (S)	0.23	(0.04, 0.43) ^†^	0.02	0
Subgroup difference				NA	NA				n.s.	0				n.s.	0
Overall	14 (M)	0.32	(0.05, 0.59) ^‡^	0.02	75	2 (M)	0.68	(0.35, 1.02) ^†^	<0.0001	0	6 (C)	0.16	(−0.22, 0.54) ^‡^	n.s.	76
MQ level (PEDro score)															
≥7/10	10 (M)	0.48	(0.19, 0.77) ^‡^	0.001	67	2 (M)	0.68	(0.35, 1.02) ^†^	<0.0001	0	4 (C)	0.23	(−0.35, 0.82) ^‡^	n.s.	83
<7/10	4 (C)	0.05	(−0.54, 0.63) ^‡^	n.s.	82	0					2 (C)	0.02	(−0.30, 0.27) ^†^	n.s.	0
Subgroup difference				n.s.	41.3				NA	NA				n.s.	0
Participant type															
Community dweller	8 (C)	0.32	(−0.08, 0.73) ^‡^	n.s.	80	0					4 (C)	0.21	(−0.38, 0.80) ^‡^	n.s.	85
Institutionalized resident	6 (C)	0.31	(−0.03, 0.65) ^‡^	n.s.	65	2 (M)	0.68	(0.35, 1.02) ^†^	<0.0001	52	2 (C)	0.05	(−0.28, 0.37) ^†^	n.s.	0
Subgroup difference				n.s.	0				NA	NA				n.s.	0
Population area															
Americas	3 (C)	0.22	(−0.36, 0.80) ^‡^	n.s.	79	0					2 (C)	−0.10	6(−0.38, 0.19) ^†^	n.s.	0
Asia	1 (M)	1.39	(0.96, 1.82)	<0.0001	NA	0					9 (C)	0.33	(−0.43, 1.09) ^‡^	n.s.	86
Europe	9 (S)	0.17	(0.01, 0.33) ^†^	0.04	0	2 (M)	0.68	(0.35, 1.02)^†^	<0.0001	52	1 (C)	0.16	(−0.31, 0.63)	n.s.	NA
Oceania	1 (L)	0.92	(0.42, 1.42)	0.0003	NA	0					0				
Subgroup difference				<0.00001	90.5				NA	NA				n.s.	0
Control group type															
PLA-S or nonexercise	8 (M)	0.38	(0.21, 0.55) ^‡^	<0.0001	77	0					4 (C)	0.37	(−0.15, 0.89)^‡^	n.s.	78
Exercise	11 (C)	0.16	(−0.01, 0.33) ^†^	n.s.	0	2 (M)	0.68	(0.35, 1.02) ^†^	<0.0001	52	5 (C)	0.10	(−0.51, 0.71) ^‡^	n.s.	84
PS	3 (S)	0.44	(0.16, 0.72) ^†^	0.002	60	0					2 (C)	0.59	(−0.27, 1.46) ^‡^	n.s.	80
Subgroup difference				n.s.	40.5				NA	NA				n.s.	0
Supplementation dose															
≥30 g/day (g/session)	8 (S)	0.26	(0.07, 0.45) ^†^	0.007	46	0					2 (C)	0.05	(−0.28, 0.37) ^†^	n.s.	0
<30 g/day (g/session)	6 (C)	0.39	(−0.13, 0.91) ^‡^	n.s.	87	2 (M)	0.68	(0.35, 1.02) ^†^	<0.0001	52	4 (M)	0.21	(−0.38, 0.80) ^‡^	n.s.	85
Subgroup difference				n.s.	0				NA	NA				n.s.	0
Exercise type															
RET	6 (C)	0.31	(−0.02, 0.64) ^‡^	n.s.	58	1 (L)	0.67	(0.30, 1.04)	0.0003	NA	2 (C)	0.05	(−0.28, 0.37)^†^	n.s.	0
MET	8 (C)	0.32	(−0.09, 0.73) ^‡^	n.s.	82	1 (C)	0.75	(−0.09, 1.58)	n.s.	NA	4 (C)	0.21	(−0.38, 0.80)^‡^	n.s.	85
Subgroup difference				n.s.	0				n.s.	0				n.s.	0
Intervention duration															
<12 weeks	6 (S)	0.32	(0.11, 0.53) ^†^	0.003	0	1 (L)	0.67	(0.30, 1.04)	0.0003	NA	1 (C)	−0.06	(−0.51, 0.40)^‡^	n.s.	NA
12–24 weeks	8 (M)	0.53	(0.09, 0.97) ^‡^	0.02	83	1 (C)	0.75	(−0.09, 1.58)	n.s.	NA	3 (C)	0.33	(−0.43, 1.09)^‡^	n.s.	86
≥24 weeks	6 (C)	−0.08	(−0.28, 0.12) ^†^	n.s.	0	0					2 (C)	−0.02	(−0.30, 0.27)^†^	n.s.	0
Subgroup difference				0.006	80.8				n.s.	0				n.s.	48.7

^†^ Fixed-effects model. ^‡^ Random-effects model. ^a^ Global frailty score is defined as a number out of five frailty components of Fried’s criteria [24]. ^b^ f evidence: strong (S), moderate (M), limited (L), conflicting (C). LoE, level of evidence; SMD, standard mean difference; *I*^2^, heterogeneity; MQ, methodological quality; PEDro, Physiotherapy Evidence Database; n.s., nonsignificant (*p* > 0.05); PLA-S, placebo supplement; PS, protein supplementation; RET, resistance exercise training; MET, multicomponent exercise training; NA, not applicable.

**Table 4:** Summary of overall effects and subgroup analysis results for body composition.

Subgroups	Lean Body Mass					Appendicular Skeletal Muscle Mass					Fat Mass				
	Comparison, *n* (LoE) ^a^	SMD	(95%CI)	*p* Value	*I*^2^ (%)	Comparison, *n* (LoE) ^a^	SMD	(95%CI)	*p* Value	*I*^2^ (%)	Comparison, *n* (LoE) ^a^	SMD	(95%CI)	*p* Value	*I*^2^ (%)
Overall	7 (S)	0.52	(0.33, 0.71) ^†^	<0.00001	51	3 (S)	0.64	(0.34, 0.93) ^†^	<0.0001	0	2 (C)	0.08	(−0.33, 0.50) ^†^	n.s.	0
MQ level (PEDro score)															
≥7/10	5 (S)	0.40	(0.17, 0.62) ^†^	0.0005	53	3 (S)	0.64	(0.34, 0.93) ^†^	<0.0001	0	2 (C)	0.08	(−0.33, 0.50) ^†^	n.s.	0
<7/10	2 (M)	0.80	(0.45, 1.14) ^†^	<0.00001	0	0					0				
Subgroup difference				n.s.	42.8				NA	NA				NA	NA
Participant type															
Community dweller	5 (S)	0.51	(0.28, 0.74) ^†^	<0.0001	54	3 (S)	0.64	(0.34, 0.93) ^†^	<0.0001	0	2 (C)	0.08	(−0.33, 0.50) ^†^	n.s.	0
Institutionalized resident	2 (M)	0.53	(0.20, 0.86) ^†^	0.002	71	0					0				
Subgroup difference				n.s.	0				NA	NA				NA	NA
Population area															
Americas	1 (C)	0.24	(−0.21, 0.69)	n.s.	NA	0					0				
Asia	2 (M)	0.80	(0.45, 1.14) ^†^	<0.00001	0	1 (M)	0.54	(0.14, 0.94)	0.009	NA	0				
Europe	4 (S)	0.45	(0.19, 0.71) ^†^	0.0007	62	2 (S)	0.75	(0.32, 1.18) ^†^	0.0006	0	2 (C)	0.08	(−0.33, 0.50) ^†^	n.s.	0
Oceania	0					0					0				
Subgroup difference				n.s.	45.6				n.s.	0				NA	NA
Control group type															
PLA-S or nonexercise	5 (M)	0.59	(0.12, 1.07) ^‡^	0.01	72	1 (M)	0.58	(0.09, 1.07)	0.02	NA	0				
Exercise	6 (S)	0.51	(0.27, 0.75) ^†^	<0.0001	42	3 (S)	0.59	(0.26, 0.91) ^†^	0.0004	0	2 (C)	0.08	(−0.33, 0.50) ^†^	n.s.	0
PS	3 (S)	0.35	(0.04, 0.66) ^†^	0.02	44	1 (M)	0.67	(0.17, 1.17)	0.009	NA	0				
Subgroup difference				n.s.	0				n.s.	0				NA	NA
Supplementation dose															
≥30 g/day (g/session)	4 (S)	0.71	(0.40, 1.01)^†^	<0.00001	0	2 (S)	0.75	(0.32, 1.18) ^†^	0.0006	0	2 (C)	0.08	(−0.33, 0.50) ^†^	n.s.	0
<30 g/day (g/session)	3 (M)	0.54	(0.03, 104)^†^	n.s.	45	1 (C)	0.37	(−0.11, 0.86)	n.s.	NA	0				
Subgroup difference				0.04	76.7				n.s.	24.4				NA	NA
Exercise type															
RET	4 (S)	0.61	(0.35, 0.88)^†^	<0.00001	34	2 (S)	0.75	(0.32, 1.18) ^†^	0.0006	0	2 (C)	0.08	(−0.33, 0.50) ^†^	n.s.	0
MET	3 (C)	0.49	(−0.02, 1.01) ^‡^	n.s.	70	1 (C)	0.37	(−0.11, 0.86)	n.s.	NA	0				
Subgroup difference				0.04	75.3				n.s.	24.4				NA	NA
Intervention duration															
<12 weeks	1 (C)	0.24	(−0.21, 0.69)	n.s.	NA	0					0				
12–24 weeks	5 (M)	0.64	(0.22, 1.06) ^‡^	0.003	67	3 (S)	0.39	(0.10, 0.68) ^†^	0.008	0	2 (C)	0.08	(−0.33, 0.50) ^†^	n.s.	0
≥24 weeks	5 (S)	0.51	(0.18, 0.84) ^†^	0.002	48	2 (S)	0.75	(0.32, 1.18) ^†^	0.0006	0	2 (S)	0.64	(0.22, 1.07) ^†^	0.003	0
Subgroup difference				n.s.	0				n.s.	46.5				n.s.	70.9

^†^ Fixed-effects model. ^‡^Random-effects model. ^a^ Level of evidence: strong (S), moderate (M), limited (L), very limited (V), conflicting (C). LoE, level of evidence; SMD, standard mean difference; *I*^2^, heterogeneity; MQ, methodological quality; PEDro, Physiotherapy Evidence Database; n.s., nonsignificant (*p* > 0.05); PLA-S, placebo supplement; PS, protein supplementation; RET, resistance exercise training; MET, multicomponent exercise training; NA, not applicable.

**Table 5:** Summary of overall effects and subgroup analysis results for physical function

Subgroups	Leg Muscle Strength					Chair Rise					Timed Up-and-Go				
	Comparison, *n* (LoE) ^a^	SMD	(95%CI)	*p* Value	*I*^2^ (%)	Comparison, *n* (LoE) ^a^	SMD	(95%CI)	*p* Value	*I*^2^ (%)	Comparison, *n* (LoE) ^a^	SMD	(95%CI)	*p* Value	*I*^2^ (%)
Overall	15 (S)	0.37	(0.23, 0.51) ^†^	<0.00001	37	9 (S)	0.30	(0.11, 0.49) ^†^	0.002	25	5 (S)	0.26	(0.02, 0.51) ^†^	0.04	32
MQ level (PEDro score)															
≥7/10	12 (S)	0.38	(0.16, 0.60) ^‡^	0.0006	50	4 (S)	0.32	(0.06, 0.59) ^†^	0.02	44	4 (C)	0.25	(−0.00, 0.50) ^†^	n.s.	46
<7/10	3 (M)	0.39	(0.08, 0.70) ^†^	0.02	20	5 (M)	0.27	(0.01, 0.54) ^†^	0.04	23	1 (C)	0.57	(−0.69, 1.83)	n.s.	NA
Subgroup difference				n.s.	0				n.s.	0				n.s.	0
Participant type															
Community dweller	9 (S)	0.23	(0.05, 0.41) ^†^	0.01	0	5 (C)	0.22	(−0.11, 0.55) ^‡^	n.s.	45	3 (C)	0.25	(−0.03, 0.53) ^†^	n.s.	64
Institutionalized resident	6 (S)	0.57	(0.36, 0.78) ^†^	<0.00001	44	4 (M)	0.39	(0.08, 0.70) ^†^	0.01	0	2 (C)	0.29	(.23, 0.82) ^†^	n.s.	0
Subgroup difference				0.01	83.2				n.s.	0				n.s.	0
Population area															
Americas	2 (S)	1.02	(0.65, 1.40) ^†^	<0.00001	0	0					1 (C)	0.23	(−0.34, 0.81)	n.s.	NA
Asia	3 (S)	0.28	(−0.09, 0.64) ^†^	n.s.	39	0					3 (C)	0.25	(0.03, 0.53) ^†^	n.s.	64
Europe	10 (S)	0.28	(0.11, 0.45) ^†^	0.001	0	8 (S)	0.31	(0.10, 0.51) ^†^	0.003	34	1 (C)	0.57	(−0.69, 1.83)	n.s.	NA
Oceania	0					1 (C)	0.25	(−0.26, 0.75)	n.s.	NA	0				
Subgroup difference				0.002	84.5				n.s.	0				n.s.	0
Control group type															
PLA-S or nonexercise	7 (S)	0.45	(0.24, 0.67) ^†^	<0.0001	37	6 (S)	0.42	(0.19, 0.65) ^†^	0.0003	31	1 (C)	0.84	(0.33, 1.34)	0.001	NA
Exercise	13 (S)	0.20	(0.04, 0.36) ^†^	0.01	39	5 (S)	0.09	(−0.19, 0.36) ^†^	n.s.	12	5 (C)	−0.01	(−0.27, 0.25) ^†^	n.s.	0
PS	4 (S)	0.30	(0.02, 0.59) ^†^	0.03	50	0					1 (M)	1.30	(0.77, 1.84)	<0.00001	NA
Subgroup difference				n.s.	20.4				n.s.	61.4				<0.0001	91.3
Supplementation dose															
≥30 g/day (g/session)	8 (M)	0.40	(0.21, 0.59) ^‡^	0.008	52	5 (S)	0.35	(0.10, 0.59) ^†^	0.005	42	0				
<30 g/day (g/session)	7 (S)	0.34	(0.15, 0.53) ^†^	0.0004	22	4 (C)	0.34	(0.15, 0.53) ^†^	n.s.	12	5 (S)	0.26	(0.02, 0.51) ^†^	0.04	32
Subgroup difference				n.s.	0				n.s.	0				NA	NA
Exercise type															
RET	6 (M)	0.37	(0.04, 0.70) ^‡^	0.03	57	4 (S)	0.29	(0.03, 0.56) ^†^	0.03	50	0				
MET	9 (S)	0.37	(0.17, 0.58) ^†^	0.0004	24	5 (S)	0.31	(0.03, 0.58) ^†^	0.03	16	5 (S)	0.26	(0.02, 0.51) ^†^	0.04	32
Subgroup difference				n.s.	0				n.s.	0				NA	NA
Intervention duration															
<12 weeks	5 (M)	0.58	(0.14, 1.01) ^‡^	0.009	60	3 (C)	−0.03	(−0.42, 0.36) ^†^	n.s.	0	4 (C)	0.06	(−0.25, 0.37) ^†^	n.s.	0
12–24 weeks	11 (S)	0.22	(0.06, 0.37) ^†^	0.006	10	6 (M)	0.23	(0.01, 0.45) ^†^	0.04	8	4 (M)	0.27	(0.01, 0.55) ^‡^	0.04	49
≥24 weeks	5 (C)	0.21	(−0.03, 0.45) ^†^	n.s.	0	5 (S)	0.14	(−0.35, 0.63) ^‡^	n.s.	75	0				
Subgroup difference				n.s.	16.7				n.s.	0				n.s.	0

^†^ Fixed-effects model. ^‡^ Random-effects model. ^a^ Level of evidence: strong (S), moderate (M), limited (L), very limited (V), conflicting (C). LoE, level of evidence; SMD, standard mean difference; *I*^2^, heterogeneity; MQ, methodological quality; PEDro, Physiotherapy Evidence Database; n.s., nonsignificant (*p* > 0.05); PLA-S, placebo supplement; PS, protein supplementation; RET, resistance exercise training; MET, multicomponent exercise training; NA, not applicable.

**Table 6:** Summary of effects of protein supplementation combined with exercise training on clinical outcomes for frail older people

Measures	PS Plus Exercise Training		PS plus MET		PS Plus RET		Influence Factors
	Effect Size (SMD) ^a^	LoE ^b^	Effect Size (SMD) ^a^	LoE ^b^	Effect Size (SMD) ^a^	LoE ^b^	
Frailty index ^c^							
Whole body mass	Large (0.44)	S	Medium (0.38)	M	Large (0.44)	S	Supplementation dose
Handgrip strength	Medium (0.26)	S	n.s.	C	Medium (0.26)	S	none
Walking speed	Medium (0.32)	M	n.s.	C	n.s.	C	Intervention duration Population area
Exhaustion	Large (0.68)	M	n.s.	C	Large (0.67)	L	none
Physical activity	n.s.	C	n.s.	C	n.s.	C	none
Global frailty score	Medium (0.26)	M	Large (0.62)	M	No evidence		none
Body composition							
LBM	Large (0.52)	S	n.s.	C	Large (0.61)	S	Supplementation dose Exercise type
ASM	Large (0.64)	S	n.s.	C	Large (0.75)	S	none
Leg strength	Medium (0.37)	S	Medium (0.37)	S	Medium (0.37)	M	Participant type; population area
Mobility							
Chair rise	Medium (0.30)	S	Medium (0.31)	S	Medium (0.29)	S	none
TUG	Medium (0.26)	S	Medium (0.26)	S	No evidence		none

^a^ Effect size: trivial (SMD < 0.10), small (0.10 ≤ SMD < 0.25), medium (0.25 ≤ SMD < 0.40), and large (SMD ≥ 0.40). ^b^ Level of evidence (LoE): strong (S), moderate (M), limited (L), conflicting (C). ^c^ Measures of the frailty index are derived from the Fried criteria [24]; global frailty score is defined as a number out of five frailty components of Fried’s criteria. ASM, appendicular skeletal muscle mass; LBM, lean body mass; LoE, level of evidence; MET, multicomponent exercise training; n.s., nonsignificant (*p* > 0.05); RET, resistance exercise training; PS, protein supplementation; SMD, standard mean difference; TUG, timed up-and-go.

## Supplementary Materials

The following are available online at http://www.mdpi.com/2072-6643/10/12/1916/s1, Table S1: Database search formulas; Table S2: Summary of protein supplementation protocols in the included studies; Table S3: Summary of exercise training protocols in the included studies; Figure S1: Forest plot summarizing effects of protein supplement (PS) plus exercise training (ET) on total body mass at an overall duration and each follow-up time point; Figure S2: Forest plot summarizing effects of protein supplement (PS) plus exercise training (ET) on handgrip strength at an overall duration and each follow-up time point; Figure S3: Forest plot summarizing effects of protein supplement (PS) plus exercise training (ET) on walking speed at an overall duration and each follow-up time point; Figure S4: Forest plot summarizing effects of protein supplement (PS) plus exercise training (ET) on exhaustion at an overall duration and each follow-up time point; Figure S5: Forest plot summarizing effects of protein supplement (PS) plus exercise training (ET) on physical activity at an overall duration and each follow-up time point; Figure S6: Forest plot summarizing effects of protein supplement (PS) plus exercise training (ET) on global frailty score at an overall duration and each follow-up time point; Figure S7: Forest plot summarizing effects of protein supplement (PS) plus exercise training (ET) on lean body mass at an overall duration and each follow-up time point; Figure S8: Forest plot summarizing effects of protein supplement (PS) plus exercise training (ET) on appendicular lean mass at an overall duration and each follow-up time point; Figure S9: Forest plot summarizing effects of protein supplement (PS) plus exercise training (ET) on fat mass at an overall duration and each follow-up time point; Figure S10: Forest plot summarizing effects of protein supplement (PS) plus exercise training (ET) on leg strength at an overall duration and each follow-up time point; Figure S11: Forest plot summarizing effects of protein supplement (PS) plus exercise training (ET) on chair rise at an overall duration and each follow-up time point; Figure S12: Forest plot summarizing effects of protein supplement (PS) plus exercise training (ET) on timed up-and-go (TUG) at an overall duration and each follow-up time point; Figure S13: Forest plot summarizing effects of protein supplement (PS) plus exercise training (ET) on SPPB at an overall duration and each follow-up time point; Figure S14: Forest plot summarizing effects of protein supplement (PS) plus exercise training (ET) on activities of daily living (ADL) at an overall duration and each follow-up time point.
