# Supplementary material for: Effects of Protein Supplementation Combined with Exercise Intervention on Frailty Indices, Body Composition, and Physical Function in Frail Older Adults
Source: Nutrients. 2018 Dec 4;10(12):1916. doi: 10.3390/nu10121916 (PMC6315527; doi:10.3390/nu10121916)
Supplement: Supplementary file 1 [file nutrients-10-01916-s001.pdf]

Table S1. Database search formulas

Data base	Search terms for query
**Pubmed**	
#1	(((frailty/) OR frail) OR elderly) OR order adults
#2	[(nursing home) OR (institution)] residents
#3	(((progressive resistance training) OR resistance exercise) OR strength training) OR strengthening exercise
#4	(((weight training) OR weight lifting/) OR weighted exercise
#5	multicomponent exercise
#6	(physical activity exercise) OR function training
#7	[(whey protein) Or (amino-acid) OR (leucine)] supplement
#8	[(diet) OR (nutrient)] intervention
#9	[(nutrient) OR (nutrition)] supplement
#10	(#1) OR 2
#11	(((#3) OR #4) OR #5) OR #6
#12	((#7) OR #8) OR #9
#13	((#10) AND #11) AND #12
**Physiotherapy Evidence Database (PEDro)**	
	Method: clinical trial
	Abstract & Title:
#1	frail elderly
#2	frailty
#3	resistance training
#4	strengthening exercise
#5	multicomponent exercise
#6	physical activity exercise
#7	protein supplement
#8	whey protein supplement

(continued)

Table S1. (continued)

Data base	Search terms for query
**Excerpta Medica dataBASE (EMBASE)**	
#1	frailty
#2	frail
#3	elderly
#4	Older adults
#5	('nursing'/exp OR nursing) AND ('home'/exp OR home)
#6	'institutional care'
#7	#1 OR #2 OR #3 OR #4 OR #5 OR #6
#8	Resistance AND training OR exercise
#9	strength AND training OR exercise
#10	strengthening exercise
#11	multicomponent AND exercise
#12	physical AND activity AND exercise AND training
#13	function training
#14	#8 OR #9 OR #10 OR #11 OR #12 OR #13
#15	whey protein supplement
#16	whey protein
#17	leucine
#18	nutrition AND supplement
#19	nutrient AND supplement
#20	#15 OR #16 OR #17 OR #18 OR #19
#33	#7 AND #14 AND #20
#34	#33 AND [randomized controlled trial]/lim AND ([article]/lim OR [article in press]/lim) AND [humans]/lim
**Cochrane Library Database**	
#1	frailty
#2	frail elderly
#3	resistance training
#4	strengthening exercise
#5	multicomponent exercise
#6	physical activity exercise
#7	protein supplement
#8	#1 OR #2
#9	#3 OR #4 OR #5 OR #6
#10	#7 AND #8 AND #9

(continued)

Table S1. (continued)

Data base	Search terms for query
**China knowledge resource integrated database**	
#1	(frailty) OR (frail elderly)
#2	exercise training
#3	(whey protein) OR (leucine)
#4	#1 AND #2 AND #3
**Google Scholar**	
#1	allintitle: frail elderly OR frailty
#2	allintitle: resistance training OR strengthening exercise
#3	allintitle: multicomponent exercise OR physical activity exercise
#4	allintitle: protein supplement OR whey OR leucine

Table S2. Guidelines of evidence synthesis[a]

Level of evidence	Criterion of judgment
Strong	Provided by consistent[b] statistically significant (or nonsignificant) pooled results in SMD or derived from multiple RCTs, including at least two high-quality RCTs[c]
Moderate	Provided by statistically significant results in one high-quality RCT[c] ***or***<br>Provided by inconsistent[b] statistically significant pooled results in SMD or derived from multiple RCTs, including at least one high-quality RCT[c] ***or***<br>Provided by consistent[b] statistically significant (or nonsignificant) pooled results in SMD or derived from multiple medium-quality RCTs[c].
Limited	Provided by statistically significant results in one medium-quality RCT[c] ***or***<br>Provided by inconsistent[b] statistically significant pooled results in SMD or derived from multiple medium-quality RCTs[c].
Conflicting	Provided by inconsistent[b] statistically nonsignificant results in SMD or derived from multiple RCTs regardless of quality

RCT = randomized controlled trial; SMD = standard mean difference; OR = odds ratio.

[a]Established in accordance with the "best-evidence synthesis," adapted by Dorrestijn et al [31] from van Tulder's criteria [32].

[b]Pooled results are considered consistent if no statistically significant heterogeneity (I^2, $P > 0.05$) is identified and inconsistent if statistically significant I^2 ($P < 0.05$) is identified.

[c]Methodological quality of a study is rated on the basis of the PEDro score as high (≥7/10) and medium (<7/10).

Table S3. Summary of protein supplementation protocols in the included studies

Study author (year) [reference number]	Experimental group					Control group
	Protein sources	Intake amount (g/d or g/session)	Weekly servings	Supplement type	Intake timing	Source of supplement
Beck 2016 [41]	Protein	18 g /session	2	Nutritional drinks in different flavors (125 ml/serving; 14.4 g protein/100 mL)	After exercise training session	No nutrition supplement
Beck 2008 [26]; 2010 [42]	Protein	7 g/d	7	Chocolate (1.3 g protein/serving); Hot chocolate (5.7 g protein/serving) or a milk-based oral supplement (6.9 g protein/serving)	Daily in the afternoon and between meals in the evening	No placebo supplement; Normal nutritional care, including oral supplements
	Milk protein	3 g/session	2	Cream and cocoa milk (3.1 g of protein/100 g); Gratin-diets were provided for residents with chewing and swallowing problems.	After exercise training session	
Bonnefoy 2003 [43]	Proteins	30 g/d (2 servings/d)	14	Nutritional energy drinks (4 different flavors; 15 g protein/serving)	Daily at 10:00 AM and 16:00 PM	Placebo supplement with an identical packaging containing neither energy and protein nor vitamins and minerals (4 different flavors).
Carlsson 2011 [44]	Milk protein	7.4 g /session	2–3	Milk-based protein-enriched drink (7.4 g protein/serving)	After exercise training session	Placebo drink (0.2 g protein/serving)
Chin A Paw 2001 [45]	Proteins	20 g/d	7	1 dairy product (100g of vanilla custard and fruit yogurt, 75g of vanilla fruit soft curd cheese)	Daily diet	Placebo supplement

Continued

Table S3. Continued.

Study author (year) [reference number]	Experimental group					Control group
	Protein sources	Intake amount (g/d or g/session)	Weekly servings	Supplement type	Intake timing	Source of supplement
Corcoran 2017 [46]	Milk protein	20 g /session	7	Nutrition supplement drink (20 g protein/serving)	After the completion of each exercise class	No nutrition supplement
de Jone 1999 [47]	Proteins	20 g/d	7	100-g servings of dairy products (vanilla custard, two types of fruit yogurt and 75 g of cheese curd with fruits)	Daily diet	Regular dairy products (highest 15% of the concentration in enriched products)
Dirks 2017 [48]	Milk protein	30 g/d (2 servings/d)	14	Beverage protein drink (15 g protein/serving)	twice daily after breakfast and after lunch	Placebo supplement (7.13 g lactose and 0.42 g calcium per serving)
Fiatarone 1994 [49]	Soy protein	40.8 g/d	7	Liquid supplement (240 ml/serving, 17% protein per serving)	Once daily in the evening	Placebo supplement
Franzke 2015 [50]	Whey protein, Leucine, EAA	20.7 g/d	9	Liquid supplement (20.7 g protein/serving; 19.7 g whey protein, 3 g leucine, ＞10 g EAA)	Once daily in the morning	No nutrition supplement
		41.4 g/session	2	Nutrient supplement drink (20.7 g protein/serving: 3 g leucine, ＞10 g EAA)	After exercise training session	No nutrition supplement
Hofmann 2016 [51]	Leucine, EAA	20.7 g/d	2	Nutrient supplement drink (20.7 g protein/serving: 3 g leucine, ＞10 g EAA)	Once daily in the morning	No nutrition supplement
		41.4 g/session	2	Nutrient supplement drink (20.7 g protein/serving: 3 g leucine, ＞10 g EAA)	After exercise training session	No nutrition supplement
Ikeda 2016 [52]	EAA	6 g/session	2	6-g amino acid supplement drink (500 mg of amino acids per 1 g, 3 g EAA/serving)	Within 10 min before exercise	Placebo supplement (6 g of maltodextrin)

Continued

Table S3. Continued.

Study author (year) [reference number]	Experimental group					Control group
	Protein sources	Intake amount (g/d or g/session)	Weekly servings	Supplement type	Intake timing	Source of supplement
Imaoka 2016 [53]	Proteins	4.1 g/d	7	Isocal jelly PCF (4 g protein/serving); Nutrition supplement (0.1 g protein/serving)	Jelly: daily after lunch; Supplement: daily after dinner	No nutrition supplement
Kim 2015 [54]	Milk protein (MFGM)	22 g/d	7	Pill-form yogurt-flavored supplement (21.5% protein, 167 mg of MFGM per pill, 6 pills/serving)	Daily in the morning	Placebo supplement (milk powder, 26.3% protein)
		22 g/session	2		Before activities	
Niccoli 2017 [55]	Whey protein	24 g/d	7	An oral dietary product	Daily; hot cereal (9 g at breakfast); milk products (7.5 g/drink at lunch and dinner)	Standard care
Oesen 2015 [56]	Leucine, EAA	20.7 g/d;	9	Nutrient supplement drink (20.7 g protein/serving: 3 g leucine, ＞10 g EAA)	Once daily in the morning;	No nutrition supplement
		41.4 g/session	2	Nutrient supplement drink (20.7 g protein/serving: 3 g leucine, ＞10 g EAA)	After exercise on the training session day	No nutrition supplement
Rosendahl 2006 [57]	Milk protein	7.4 g/session	2–3	Nutrient supplement drink (7.4 g protein, 15.7 g carbohydrate)	After exercise on the training session day	Placebo supplement (0.2 g protein, 10.8 g carbohydrate)

Continued

Table S3. Continued.

Study author (year) [reference number]	Experimental group					Control group
	Protein sources	Intake amount (g/d or g/session)	Weekly servings	Supplement type	Intake timing	Source of supplement
Tieland 2012 [58]	Milk protein	30 g/d	14 (2 serving/d)	A 250-mL protein-supplemented beverage (vanilla flavored milk protein concentrate; 15 g protein/serving)	Daily, 1 beverage consumed after breakfast and 1 beverage consumed after lunch	Placebo supplement (7.1 g lactose, 0.4 g calcium)
Trabal 2015 [59]	Leucine	10 g/d	15	5 g leucine (about 40 g of whey protein) per serving	Twice daily after main meals (lunch and dinner)	Placebo supplement (maltodextrin)
Yamada 2015 [60]	Protein (BCAA)	10 g/d	7	Protein supplement (10.0 g of protein with BCAA per serving)	Daily	No nutrition supplement
Zak 2009 [61]	Protein	32 g/d	7	A liquid supplement drink (200 ml/serving; 16% protein)	Once daily, shortly before the commencement of their routine exercise regimen	Placebo supplement (200 ml/serving; 97.5% carbohydrates, 1% lipids, 1.5% proteins)

BCAA, branched chain amino acids; EAA, essential amino acids; MFGM, milk fat globule membrane

Table S4. Summary of exercise training protocols in the included studies

Study author (year) [reference number]	Experimental group											Control group
	Flexibility/ROM/stretching exercises	Muscle strengthening (progressive RET)				Endurance (AET)	Balance	Functional mobility exercises	Training time (min/session)	Frequency (session/w)	Intervention duration (wk)	Control activity
		Training part	Resistance set	Intensity	Training volume							
Beck 2016 [41]	None	Upper and lower extremity exercises and weight bearing exercises	Light weights, theraband or body resistance	Moderate	NR	None	Included; No detailed information.	Transfers, bed mobility, gait/wheelchair training, stair training.	30–45	2	11	Standard care from nutrition coordinators, physiotherapists, and occupational therapists.
Beck 2008 [26]; 2010 [42]	Included as warm-up exercise.	Upper and lower extremity functional strength training	Weight bearing	Moderate	NR	None	Dynamic balance training (weight shifts in the sitting and standing positions, walking on different surfaces, and ball bounces)	None	45–60	2	11	Standard care from nutrition coordinators, physiotherapists, and occupational therapists.
Bonnefoy 2003 [43]	Flexibility exercise, included as warm-up exercise.	Upper and lower extremity exercises	Dumbbells, elastic bands, and weight bearing exercises	NR	Started with one set of five repetitions and progressed to three sets of ten.	None	Included; No detailed information.	Group training (ball-games) incorporated with strengthening and standing exercises.	60	3	36	NR
Carlsson 2011 [44]	None	Lower limbs	Weight bearing	High (8-12 RM); 18-15 RM for the first two weeks (build-up period)	12 repetitions; 2 sets (at least two lower-limb strength exercises and two balance exercises)	None	Balance exercises while standing and walking	Functional weight-bearing exercise	45	2–3	13	Theme-based sitting activities (watching films, reading, singing, conversation)
Chin A Paw 2001 [45]	Cool-down period consisted of stretching and relaxation activities (eg, finger and wrist rolls, shoulder rolls, reaching, leg stretches)	Lower extremity	Wrist and ankle weights (450g each)	Moderate; 6–8 on a 10-point RPE scale (1 = very, very light, 10 = very, very heavy)	NR	Included; No detailed information.	Coordination; motor behavior in games and cooperative activities (ie, throwing, catching a ball) while standing up and sitting down on a chair, musical chairs, and team pursuit races	Group training; Walking (warm-up activities); Motor actions (reaching, throwing, catching, kicking, chair stands, bending down, toe and heel raises)	45	2	17	Social program (lectures, social activities, crafts; once every 2 weeks for 90 minutes); Home visits (a supply of fresh food products; off weeks)

Continued

Table S4. Continued.

Study author (year) [reference number]	Experimental group: Flexibility/ROM/stretching exercises	Muscle strengthening (progressive RET): Training part	Muscle strengthening (progressive RET): Resistance set	Muscle strengthening (progressive RET): Intensity	Muscle strengthening (progressive RET): Training volume	Endurance (AET)	Balance	Functional mobility exercises	Training time (min/session)	Frequency (session/w)	Intervention duration (wk)	Control group: Control activity
Corcoran 2017 [46]	None	Lower extremity (chair stands, leg extensions, knee flexions, and hip abductions)	Ankle weight (0 to 5 pounds/leg); body weight	RPE: somewhat hard to moderately hard	10 repetitions; 2 sets	Marching or dancing (progressed in duration (15 to 30 minutes),); speed of movements based on music cadence (90 beats/minute (bpm), 100 bpm, 110 bpm)	tandem stands, crossover walks, toe stands, vestibular-based exercises (standing on one leg, turning one's head side to side)	None	60	3	24	An attention-control program (once/week discussion group for 60 minutes)
de Jone 1999 [47]	Included; No detailed information.	Lower extremity	Weights, elastic bands	Progressively increasing intensity	NR	Included; No detailed information.	Coordination; exercise using balls and ropes	walking, stooping, chair stands; increasing daily activity level	45	2	17	Social program (attention; once every 2 weeks for 90 minutes)
Dirks 2017 [48]	Cycle ergometer (warm-up & cool-down)	Whole body	Leg press and leg extension machine	50%–75% 1-RM	Initial session: 10-15 repetitions; leg press and leg extension machine (4 sets); 3 sets on the lateral pulldown, vertical row, chest press, and pec (pectoral) dec. Advance (4 wks later): 75% of 1RM for 8 repetitions on leg press and leg extension	None	None	None	NR	2	24	The same exercise program as exponential group did.
Fiatarone 1994 [49]	None	Lower extremity	Leg press and leg extension machine	80% 1-RM	6-9 sec/repetition; 3 sets, 8 repetitions	None	None	None	45	3	10	Placebo activities: walking, calisthenics in seated position, board games, crafts, concerts, group discussions

Continued

Table S4. Continued.

Study author (year) [reference number]	Experimental group											Control group
	Flexibility/ROM/stretching exercises	Muscle strengthening (progressive RET)				Endurance (AET)	Balance	Functional mobility exercises	Training time (min/session)	Frequency (session/w)	Intervention duration (wk)	Control activity
		Training part	Resistance set	Intensity	Training volume							
Franzke 2015 [50]	None	Whole body: ten exercises for the main muscle groups (legs, back, abdomen, chest, shoulder and arms)	Elastic bands	OMNI-RES > 7 (80% 1-RM)a	Initial phase (first 4 weeks): one set of 15 repetitions; Advance phase (after 5th wk): 2 sets of 15 repetitions	None	None	None	60	2	24	RET group: The same as experimental group did. Cognitive training group: memory training and finger dexterity exercises in sitting position.
Hofmann 2016 [51]	None	Whole body: ten exercises for the main muscle groups (legs, back, abdomen, chest, shoulder and arms)	Elastic bands	OMNI-RES > 7 (80% 1-RM)	Initial phase (first 4 weeks): one set of 15 repetitions; Advance phase (after 5th wk): 2 sets of 15 repetitions	None	None	None	60	2	24	Cognitive training group (placebo activity): cognitive tasks (memory training) and coordinative tasks (such as manual dexterity), 2 session/wk; RET control group: The exercise program as experimental group did.
Ikeda 2016 [52]	10 min, cool-down	Lower extremity	Muscle training machine	30% 1-RM	3 sets, 20 repetitions	1 set, 10 min; ergometer or a recumbent cross trainer; RPE level: 12	1 set, 15 min; balance pad	gait training in the parallel bars	110	2	12	The same exercise program as experimental group did.
Imaoka 2016 [53]	Group exercise: warm-up and cool-down activities	Upper and lower extremity (Individualized and group exercise)	Elastic bands (Individualized and group exercise)	NR	NR	None	Sitting and standing balance exercise (Individualized and group exercise)	Individualized exercise: Transfers, gait, sit-to-stand exercise; Group exercise: sit-to-stand exercise (20 repetitions, 1 set)	Individualized exercise: 20 min; Group exercise: 30 min	Individualized exercise: 2 session/wk; Group exercise: 1 session/wk)	12	Usual care: individualized exercise (20 min, 2 session/wk), group exercise (30 min, 1 session/wk)

Continued

Table S4. Continued.

Study author (year) [reference number]	Experimental group											Control group
	Flexibility/ROM/stretching exercises	Muscle strengthening (progressive RET)				Endurance (AET)	Balance	Functional mobility exercises	Training time (min/session)	Frequency (session/w)	Intervention duration (wk)	Control activity
		Training part	Resistance set	Intensity	Training volume							
Kim 2015 [54]	Warm-up (10 min) and cool-down (10 min) activities	Upper and lower extremity (toe raises, heel raises, knee lifts, knee extensions, hip flexions, lateral leg raises, double-arm pull downs, bicep curls)	Elastic bands (progressively from the seated to standing positions such as standing upright behind the chair and holding the back of the chair for stability)	Moderate (30 min); Borg RPE: 12–14 (60%–80% of 1-RM)[a]	NR	None	20 min; one leg and multidirectional weight shifts	Gait (20 min)	60	2	12	Exercise control group: the same exercise program as experimental group did.
Niccoli 2017 [55]	Included	Upper and lower extremity exercises	Light weights, theraband, weight bearing	NR	NR	Seated bike, arm ergometer and/or ambulating around the unit	Static and dynamic balance training in sitting and standing. Standing balance exercises (unsupported reach, tandem, unipedal stance, tandem walk, agility ladder stepping)	Transfers, bed mobility, gait/wheelchair training, stair training.	NR	7	4 wk (mean length of hospital stay)	Standard care
Oesen 2015 [56]	Warm-up (10 min) and cool-down (10 min) activities	Whole body: 1–2 of 10 exercises, mainly involved 6 muscle groups (legs, back, abdomen, chest, shoulder, arms)	Elastic bands (leg extension, hip extension, standing row, chest press, front raise, elbow flexion and extension); body weight (squat, calf lift, bilateral leg lift hold)	35–40 min; OMNI-RES > 7 (80% 1-RM)a	Initial phase (first 4 weeks): one set of 15 repetitions; Advance phase (after 5th wk): 2 sets of 15 repetitions	None	None	None	60	2	24	Cognitive training group (placebo activity): cognitive tasks (memory training) and coordinative tasks (such as manual dexterity), 2 session/wk; RET control group: The exercise program as experimental group did.

Continued

Table S4. Continued.

Study author (year) [reference number]	Experimental group											Control group
	Flexibility/ROM/stretching exercises	Muscle strengthening (progressive RET)				Endurance (AET)	Balance	Functional mobility exercises	Training time (min/session)	Frequency (session/w)	Intervention duration (wk)	Control activity
		Training part	Resistance set	Intensity	Training volume							
Rosendahl 2006 [57]	None	Lower extremity (Step-up onto boxes, heel raises, squat, sit to stand, forward or side lunge)	Body weight, weighted belt (worn around the waist)	High-intensity (8–12 RM)	Weighted belt: up to a maximum of 12 kg; Body weight: doing deeper squats or doing step-ups onto a higher box	None	Walking over obstacles, on a soft surface, with numerous turns; trunk rotation; side step and return	- After training sessions<br>- Physical tasks regarding daily life activities (e.g. walking, squats, and standing without balance support)<br>- Number: one to four<br>- Frequency: from weekly up to daily	45	2–3	13	Included activities while sitting, e.g. watching films, reading, singing, and conversation
Tieland 2012 [58]	Warm-up (5 min, cycle ergometer)	Upper and lower extremity	Leg-extension machines; chest press, lat pulldown, pec deck, and vertical row machines	Started at 50% of 1-RM (10-15 repetitions per set); Progressively increased to 75% of 1-RM (8–10 repetitions)	4 sets on the leg-press and leg-extension machines and 3 sets on chest press, lat pulldown, pec deck, and vertical row machines	None	None	None	NR	2	24	The same exercise program as experimental group did.
Trabal 2015 [59]	Warm-up (5 min) and cool-down (5 min) activities	Lower extremity (chair squats, leg curls, leg extensions, toe stands, wall push-ups)	Body weight (exercises were executed while seated or with the use of a chair as a support aid)	65% of the maximum number of repetitions	Started with 1 set of 8 repetitions, progressively increased to 2 sets of 15 repetitions	None	side leg raises, back leg raises, hip flexions, and walking heel to toe	None	RET: 40 min/session	4 (3 sessions of RET, 1 session of balance training)	12	The same exercise program as experimental group did.

Continued

Table S4. Continued.

Study author (year) [reference number]	Experimental group											Control group
	Flexibility/ROM/stretching exercises	Muscle strengthening (progressive RET)				Endurance (AET)	Balance	Functional mobility exercises	Training time (min/session)	Frequency (session/w)	Intervention duration (wk)	Control activity
		Training part	Resistance set	Intensity	Training volume							
Yamada 2015 [60]	None	None	NA	NA	NA	Pedometer-based walking programs (increase the number of daily steps by 10% each month)	None	Pedometer-based walking programs (increase the number of daily steps by 10% each month); The mail-based intervention consisted of motivation for walking followed by goal setting, self-monitoring, and feedback.	NR	7	24	The same exercise program as experimental group did.
Zak 2009 [61]a	Warm-up [5 min, upper and lower limbs; trunk (initially in an recumbent position, and in a sitting down position on a chair afterwards)]; Cool-down (simple breathing and relaxation exercises)	Lower extremity (hip extensors and flexors; knee extensors and flexors)	Elastic resistance bands (four series of resistance exercises)	High-intensity activity (80% of 1-RM)	3 sets of 10 repetitions	None	Multi-sensory balance training on a ball cushion on top of the chair seat	10 simple exercises (implemented in an upright sitting position on a standardized chair)	45 (20 min of FOE , 20 min of RET)	5	7	The same RET+FOE program as experimental group did.
Zak 2009 [61]b	Warm-up [5 min, upper and lower limbs; trunk (initially in an recumbent position, and in a sitting down position on a chair afterwards)]; Cool-down (simple breathing and relaxation exercises)	None	NA	NA	NA	Pedal exercises (duration: ca.10 min. with three 30 sec. intervals)	Multi-sensory balance training on a ball cushion on top of the chair seat	10 simple exercises (implemented in an upright sitting position on a standardized chair)	45 (20 min of FOE , 20 min of SE)	5	7	The same SE+FOE program as experimental group did.

[a]Data was estimated based on the previous studies (Ref).

AET, Aerobic exercise training; FOE, functionally-oriented exercise; OMNI-RES, OMNI-Resistance Exercise Scale (0 extremely easy to 10 extremely hard); ROM, range of motion; RM, repetition maximum; RPE, rate of perceived exertion; SE, standard exercise

Study or Subgroup	PS plus ET Mean	PS plus ET SD	PS plus ET Total	Control Mean	Control SD	Control Total	Weight	Std. Mean Difference IV, Fixed, 95% CI
**22.1.1 Overall follow-up duration, Fixed**								
Beck 2008 [26], RET+PS vs RET	1.16	11.8	62	-0.32	15.71	59	16.8%	0.11 [-0.25, 0.46]
Beck 2016 [41], MET+PS vs Con, free living	0.5	1.5	46	0.2	3.1	18	7.2%	0.14 [-0.40, 0.69]
Beck 2016 [41], MET+PS vs Con, nursing home	0.5	1.99	9	-0.36	3.89	22	3.5%	0.24 [-0.54, 1.02]
Bonnefoy 2003 [43], MET+PS vs PLA-S	2.28	3	22	-0.14	2.8	22	5.6%	0.82 [0.20, 1.44]
Carlsson 2011 [44], MET+PS vs CG	0.9	10.43	36	0.2	12.04	109	15.0%	0.06 [-0.32, 0.44]
de Jone, 1999 [47], MET+PS vs CG	0.2	0.6	39	-0.1	0.78	106	15.6%	0.41 [0.04, 0.78]
Dirks 2017 [48], RET+PS vs RET+PLA-S	2.1	10.3	17	-0.4	8.2	17	4.7%	0.26 [-0.41, 0.94]
Fiatarone 1994 [49], RET+PS vs CG	1	2	23	0.15	2.1	75	9.6%	0.41 [-0.06, 0.88]
Kim 2015 [54], MET+PS vs CG	0	28	33	-20.3	31.4	98	13.2%	0.66 [0.26, 1.06]
Tieland 2012 [58], RET+PS vs RET+PLA-S	1.8	2.12	31	-0.55	2.36	31	7.5%	1.03 [0.50, 1.57]
Trabal 2015 [59], MET+PS vs MET+PLA-S	0.571	1.33	7	-0.228	0.82	4	1.3%	0.62 [-0.65, 1.89]
**Subtotal (95% CI)**			**325**			**561**	**100.0%**	**0.38 [0.23, 0.52]**
Heterogeneity: Chi² = 15.77, df = 10 (P = 0.11); I² = 37%								
Test for overall effect: Z = 5.08 (P < 0.00001)								
**22.1.2 Whole body mass, ≥6 mo, <12 mo; Random**								
Beck 2008 [26], RET+PS vs RET	-0.65	11.8	62	-1.7	15.8	59	19.4%	0.08 [-0.28, 0.43]
Bonnefoy 2003 [43], MET+PS vs PLA-S	1.36	5.1	22	-0.53	4.6	22	14.5%	0.38 [-0.21, 0.98]
Carlsson 2011 [44], MET+PS vs CG	1.7	9.82	36	0.85	11.7	102	18.9%	0.08 [-0.30, 0.46]
Dirks 2017 [48], RET+PS vs RET+PLA-S	2.1	10.3	17	-0.4	8.2	17	13.1%	0.26 [-0.41, 0.94]
Kim 2015 [54], MET+PS vs CG	39.4	28	33	14.2	26.7	98	18.3%	0.93 [0.52, 1.34]
Tieland 2012 [58], RET+PS vs RET+PLA-S	1.8	2.12	31	-0.55	2.36	31	15.8%	1.03 [0.50, 1.57]
**Subtotal (95% CI)**			**201**			**329**	**100.0%**	**0.45 [0.09, 0.81]**
Heterogeneity: Tau² = 0.14; Chi² = 17.94, df = 5 (P = 0.003); I² = 72%								
Test for overall effect: Z = 2.44 (P = 0.01)								
**22.1.3 Whole body mass, ≥3 mo, <6 mo; Fixed**								
Bonnefoy 2003 [43], MET+PS vs PLA-S	2.28	3	22	-0.14	2.8	22	8.8%	0.82 [0.20, 1.44]
Carlsson 2011 [44], MET+PS vs CG	0.9	10.43	36	0.2	12.04	109	23.5%	0.06 [-0.32, 0.44]
de Jone, 1999 [47], MET+PS vs CG	0.2	0.6	39	-0.1	0.78	106	24.4%	0.41 [0.04, 0.78]
Dirks 2017 [48], RET+PS vs RET+PLA-S	1.3	10.29	17	0.4	8.2	17	7.4%	0.09 [-0.58, 0.77]
Kim 2015 [54], MET+PS vs CG	0	28	33	-20.3	31.4	98	20.6%	0.66 [0.26, 1.06]
Tieland 2012 [58], RET+PS vs RET+PLA-S	0.85	1.93	31	0.2	1.91	31	13.3%	0.33 [-0.17, 0.84]
Trabal 2015 [59], MET+PS vs MET+PLA-S	0.571	1.33	7	-0.228	0.82	4	2.1%	0.62 [-0.65, 1.89]
**Subtotal (95% CI)**			**185**			**387**	**100.0%**	**0.38 [0.20, 0.57]**
Heterogeneity: Chi² = 7.44, df = 6 (P = 0.28); I² = 19%								
Test for overall effect: Z = 4.12 (P < 0.0001)								
**22.1.4 Whole body mass, <3 mo; Fixed**								
Beck 2008 [26], RET+PS vs RET	0.1	3.69	62	-0.1	4.81	59	29.3%	0.05 [-0.31, 0.40]
Beck 2008 [26], RET+PS vs RET	1.16	11.8	62	-0.32	15.71	59	29.3%	0.11 [-0.25, 0.46]
Beck 2016 [41], MET+PS vs Con, free living	0.5	1.5	46	0.2	3.1	18	12.5%	0.14 [-0.40, 0.69]
Beck 2016 [41], MET+PS vs Con, nursing home	0.5	1.99	9	-0.36	3.89	22	6.2%	0.24 [-0.54, 1.02]
Fiatarone 1994 [49], RET+PS vs CG	1	2	23	0.15	2.1	75	16.8%	0.41 [-0.06, 0.88]
Trabal 2015 [59], MET+PS vs MET+PLA-S	0.63	2.82	12	0.57	3.19	12	5.8%	0.02 [-0.78, 0.82]
**Subtotal (95% CI)**			**214**			**245**	**100.0%**	**0.15 [-0.05, 0.34]**
Heterogeneity: Chi² = 1.67, df = 5 (P = 0.89); I² = 0%								
Test for overall effect: Z = 1.49 (P = 0.14)								

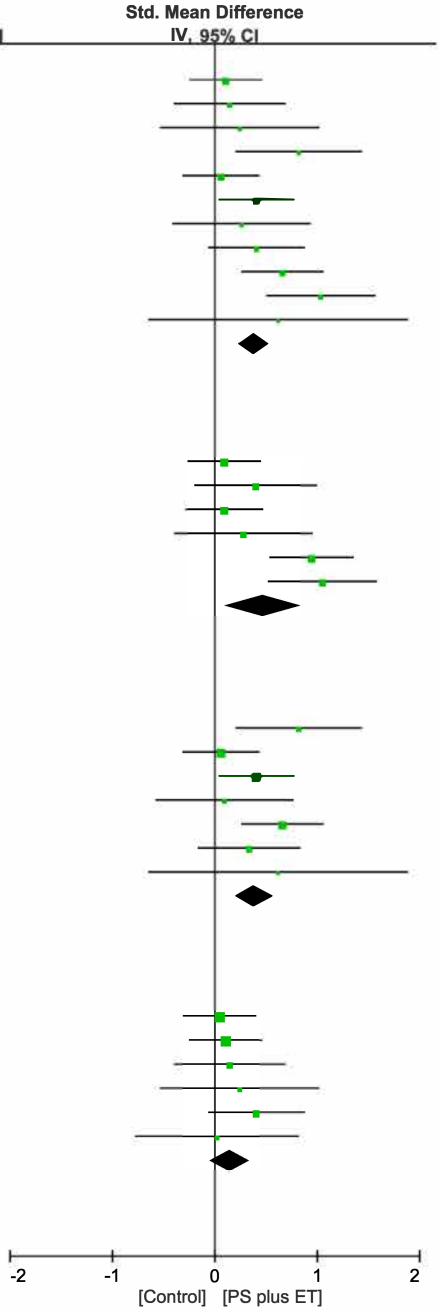

Figure S1. Forest plot summarizing effects of protein supplement (PS) plus exercise training (ET) on total body mass at an overall duration and each follow-up time point. The horizontal line links the lower and upper limits of the 95% CI of this effect. The combined effects are plotted using black diamonds. 95% CI = 95% confidence interval; Fixed = fixed-effects model; Random = random-effects model; Std. = standard; IV = inverse variance; CG = control group; Con = control; MET = multicomponent exercise training; PLA-S, placebo supplement; RET = resistance exercise training.

Study or Subgroup	PS plus ET Mean	SD	Total	Control Mean	SD	Total	Weight	Std. Mean Difference IV, Fixed, 95% CI
**22.2.1 Overall follow-up duration; Fixed**								
Beck 2008 [26], RET+PS vs RET	0.6	5.46	62	-1.2	9.49	59	12.1%	0.23 [-0.13, 0.59]
Beck 2016 [41], MET+PS vs Con, free living	-0.8	3.2	46	-0.3	4.3	18	5.2%	-0.14 [-0.69, 0.41]
Beck 2016 [41], MET+PS vs Con, nursing home	-0.2	2.9	9	-0.6	3.5	22	2.6%	0.12 [-0.66, 0.89]
Chin A Paw 2001 [45], MET+PS vs PLA-S	1	3.1	42	0.5	2.7	37	7.9%	0.17 [-0.27, 0.61]
Corcoran 2017 [46], MET+PS vs Con	1	6.1	67	0.8	6.2	54	12.0%	0.03 [-0.33, 0.39]
Dirks 2017 [48], RET+PS vs RET+PLA-S	3.5	6	17	0.5	7.4	17	3.3%	0.43 [-0.25, 1.12]
Franzke 2015 [50], RET+PS vs CG	0.3	3.5	27	0.5	4.6	45	6.8%	-0.05 [-0.52, 0.43]
Hofmann 2016 [51], RET+PS vs CG	0.09	0.14	21	0.07	0.26	49	5.9%	0.09 [-0.43, 0.60]
Ikeda 2016 [52], MET+PS vs MET+PLA-S, Tr1	-1	4	27	0.8	6.1	25	5.1%	-0.35 [-0.89, 0.20]
Ikeda 2016 [52], MET+PS vs MET+PLA-S, Tr2	0.5	5.2	25	-0.8	3	27	5.2%	0.30 [-0.24, 0.85]
Imaoka 2016 [53], MET+PS vs CG	1.64	2.9	23	2	2.8	68	6.9%	-0.13 [-0.60, 0.35]
Kim 2015 [54], MET+PS vs CG	-0.19	2.43	33	-0.69	1.83	98	9.9%	0.25 [-0.15, 0.64]
Niccoli 2017 [55], MET+PS vs MET+PLA-S	2.01	1.2	22	0.67	1.5	25	4.2%	0.96 [0.35, 1.57]
Oesen 2015 [56], RET+PS vs CG	1	2.5	25	0.03	2.5	57	6.9%	0.38 [-0.09, 0.86]
Tieland 2012 [58], RET+PS vs RET+PLA-S	2.2	2.77	31	0.45	2.8	31	5.9%	0.62 [0.11, 1.13]
**Subtotal (95% CI)**			**477**			**632**	**100.0%**	**0.17 [0.05, 0.30]**
Heterogeneity: Chi² = 19.03, df = 14 (P = 0.16); I² = 26%								
Test for overall effect: Z = 2.76 (P = 0.006)								
**22.2.2 Handgrip strength, ≥6 mo, <12 mo; Fixed**								
Corcoran 2017 [46], MET+PS vs Con	1	6.1	67	0.8	6.2	54	24.0%	0.03 [-0.33, 0.39]
Dirks 2017 [48], RET+PS vs RET+PLA-S	3.4	6.44	17	0.7	7.56	17	6.7%	0.38 [-0.30, 1.05]
Franzke 2015 [50], RET+PS vs CG	0.3	2.1	22	0.03	3.1	48	12.1%	0.09 [-0.41, 0.60]
Hofmann 2016 [51], RET+PS vs CG	0.09	0.14	21	0.07	0.26	49	11.8%	0.09 [-0.43, 0.60]
Kim 2015 [54], MET+PS vs CG	-0.19	2.43	33	-0.69	1.83	98	19.7%	0.25 [-0.15, 0.64]
Oesen 2015 [56], RET+PS vs CG	1	2.5	25	0.03	2.5	57	13.7%	0.38 [-0.09, 0.86]
Tieland 2012 [58], RET+PS vs RET+PLA-S	2.2	2.77	31	0.45	2.8	31	11.9%	0.62 [0.11, 1.13]
**Subtotal (95% CI)**			**216**			**354**	**100.0%**	**0.23 [0.05, 0.41]**
Heterogeneity: Chi² = 4.59, df = 6 (P = 0.60); I² = 0%								
Test for overall effect: Z = 2.56 (P = 0.01)								
**22.2.3 Handgrip strength, ≥3 mo, <6 mo; Fixed**								
Chin A Paw 2001 [45], MET+PS vs PLA-S	1	3.1	42	0.5	2.7	37	10.2%	0.17 [-0.27, 0.61]
Corcoran 2017 [46], MET+PS vs Con	1.4	9.5	67	1.5	9.1	54	15.5%	-0.01 [-0.37, 0.35]
Dirks 2017 [48], RET+PS vs RET+PLA-S	3.5	6	17	0.5	7.4	17	4.3%	0.43 [-0.25, 1.12]
Franzke 2015 [50], RET+PS vs CG	0.3	3.5	27	0.5	4.6	45	8.8%	-0.05 [-0.52, 0.43]
Hofmann 2016 [51], RET+PS vs CG	0.06	0.1	24	0.05	0.2	52	8.5%	0.06 [-0.43, 0.54]
Ikeda 2016 [52], MET+PS vs MET+PLA-S, Tr1	-1	4	27	0.8	6.1	25	6.6%	-0.35 [-0.89, 0.20]
Ikeda 2016 [52], MET+PS vs MET+PLA-S, Tr2	0.5	5.2	25	-0.8	3	27	6.7%	0.30 [-0.24, 0.85]
Imaoka 2016 [53], MET+PS vs CG	1.64	2.9	23	2	2.8	68	8.9%	-0.13 [-0.60, 0.35]
Kim 2015 [54], MET+PS vs CG	0.64	2.4	33	0.4	1.8	98	12.8%	0.12 [-0.27, 0.52]
Oesen 2015 [56], RET+PS vs CG	0.4	2.5	28	0.08	2.6	62	10.0%	0.12 [-0.32, 0.57]
Tieland 2012 [58], RET+PS vs RET+PLA-S	1.35	2.8	31	0	2.7	31	7.8%	0.48 [-0.02, 0.99]
**Subtotal (95% CI)**			**344**			**516**	**100.0%**	**0.09 [-0.05, 0.23]**
Heterogeneity: Chi² = 7.96, df = 10 (P = 0.63); I² = 0%								
Test for overall effect: Z = 1.20 (P = 0.23)								
**22.2.4 Handgrip strength, <3 mo; Fixed**								
Beck 2008 [26], RET+PS vs RET	0.6	5.46	62	-1.2	9.49	59	50.3%	0.23 [-0.13, 0.59]
Beck 2016 [41], MET+PS vs Con, free living	-0.8	3.2	46	-0.3	4.3	18	21.6%	-0.14 [-0.69, 0.41]
Beck 2016 [41], MET+PS vs Con, nursing home	-0.2	2.9	9	-0.6	3.5	22	10.7%	0.12 [-0.66, 0.89]
Niccoli 2017 [55], MET+PS vs MET+PLA-S	2.01	1.2	22	0.67	1.5	25	17.4%	0.96 [0.35, 1.57]
**Subtotal (95% CI)**			**139**			**124**	**100.0%**	**0.27 [0.01, 0.52]**
Heterogeneity: Chi² = 7.35, df = 3 (P = 0.06); I² = 59%								
Test for overall effect: Z = 2.06 (P = 0.04)								

Std. Mean Difference
IV, Fixed, 95% CI

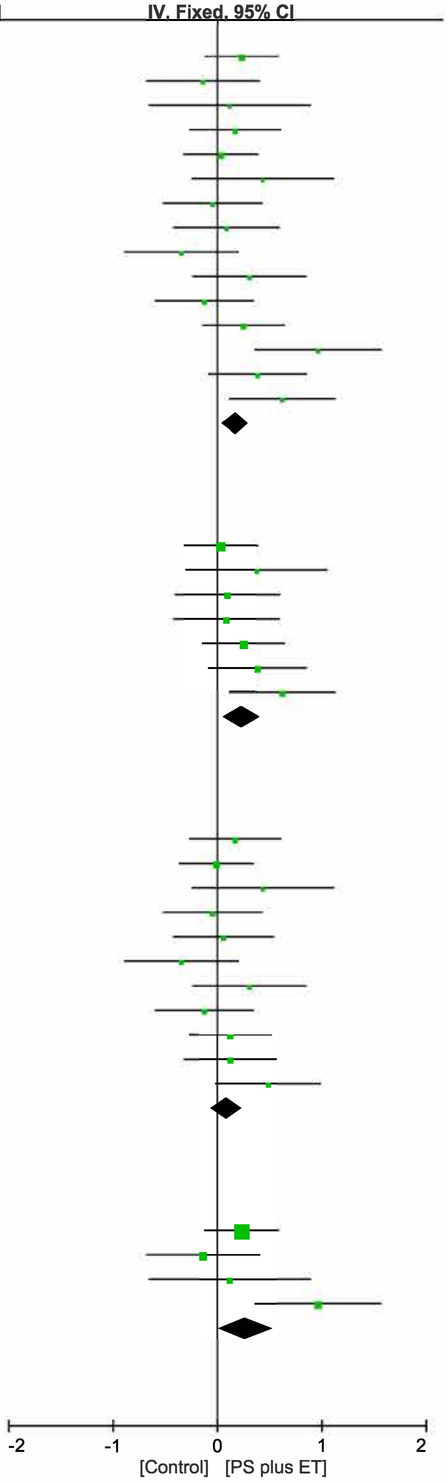

Figure S2. Forest plot summarizing effects of protein supplement (PS) plus exercise training (ET) on handgrip strength at an overall duration and each follow-up time point. The horizontal line links the lower and upper limits of the 95% CI of this effect. The combined effects are plotted using black diamonds. 95% CI = 95% confidence interval; Fixed = fixed-effects model; Std. = standard; IV = inverse variance; CG = control group; Con = control; MET = multicomponent exercise training; PLA-S, placebo supplement; RET = resistance exercise training; Tr = treatment session.

Study or Subgroup	PS plus ET Mean	PS plus ET SD	PS plus ET Total	Control Mean	Control SD	Control Total	Weight	Std. Mean Difference IV, Random, 95% CI
**22.3.1 Overall follow-up duration, Random**								
Bonnefoy 2003 [43], MET+PS vs PLA-S	20.5	27.6	22	15.8	32.2	22	6.7%	0.15 [-0.44, 0.75]
Chin A Paw 2001 [45], MET+PS vs PLA-S	0.06	0.08	42	0.01	0.09	37	7.7%	0.58 [0.13, 1.04]
Corcoran 2017 [46], MET+PS vs Con	-37	229	67	27	211	54	8.4%	-0.29 [-0.65, 0.07]
Dirks 2017 [48], RET+PS vs RET+PLA-S	-0.2	1.04	17	-0.4	0.59	17	6.1%	0.23 [-0.44, 0.91]
Fiatarone 1994 [49], RET+PS vs CG	14.9	26.7	25	2.03	26.7	75	7.7%	0.48 [0.02, 0.94]
Franzke 2015 [50], RET+PS vs CG	42	44.7	27	11.2	23.9	45	7.3%	0.92 [0.42, 1.42]
Kim 2015 [54], MET+PS vs CG	0.08	0.06	33	0.016	0.04	98	7.9%	1.39 [0.96, 1.82]
Niccoli 2017 [55], MET+PS vs MET+PLA-S	0.2	0.04	22	0.18	0.03	25	6.7%	0.56 [-0.02, 1.15]
Oesen 2015 [56], RET+PS vs CG	0	0.4	28	0.08	0.33	62	7.7%	-0.22 [-0.67, 0.22]
Rosendahl 2006 [57], MET+PS vs CG	0.02	0.12	42	-0.01	0.12	125	8.4%	0.25 [-0.10, 0.60]
Tieland 2012 [58], RET+PS vs RET+PLA-S	-0.1	0.6	31	-0.2	0.56	31	7.4%	0.17 [-0.33, 0.67]
Trabal 2015 [59], MET+PS vs MET+PLA-S	-0.62	1.56	12	-0.24	1.56	12	5.2%	-0.24 [-1.04, 0.57]
Zak 2009 [61], MET+PS vs MET+PLA-S	36.4	55.1	19	35.7	58.9	21	6.5%	0.01 [-0.61, 0.63]
Zak 2009 [61], RET+PS vs RET+PLA-S	20.7	58.8	19	2.35	55.2	21	6.4%	0.32 [-0.31, 0.94]
**Subtotal (95% CI)**			**406**			**645**	**100.0%**	**0.32 [0.05, 0.59]**
Heterogeneity: Tau² = 0.19; Chi² = 51.95, df = 13 (P < 0.00001); I² = 75%								
Test for overall effect: Z = 2.35 (P = 0.02)								
**22.3.2 Walking speed, ≥6 mo, <12 mo; Random**								
Bonnefoy 2003 [43], MET+PS vs PLA-S	20.5	27.6	22	15.8	32.2	22	11.5%	0.15 [-0.44, 0.75]
Corcoran 2017 [46], MET+PS vs Con	-37	229	67	27	211	54	13.7%	-0.29 [-0.65, 0.07]
Dirks 2017 [48], RET+PS vs RET+PLA-S	0.2	2.6	17	0.4	1.5	17	10.7%	-0.09 [-0.76, 0.58]
Franzke 2015 [50], RET+PS vs CG	39	69.1	22	31	98.8	48	12.3%	0.09 [-0.42, 0.59]
Kim 2015 [54], MET+PS vs CG	0.08	0.06	33	0.016	0.04	98	13.1%	1.39 [0.96, 1.82]
Oesen 2015 [56], RET+PS vs CG	0.12	0.46	25	0.13	0.37	57	12.7%	-0.02 [-0.49, 0.45]
Rosendahl 2006 [57], MET+PS vs CG	0	0.11	39	0.01	0.12	118	13.7%	-0.08 [-0.45, 0.28]
Tieland 2012 [58], RET+PS vs RET+PLA-S	0.1	1.91	31	0.2	1.77	31	12.4%	-0.05 [-0.55, 0.44]
**Subtotal (95% CI)**			**256**			**445**	**100.0%**	**0.14 [-0.26, 0.54]**
Heterogeneity: Tau² = 0.27; Chi² = 40.89, df = 7 (P < 0.00001); I² = 83%								
Test for overall effect: Z = 0.68 (P = 0.49)								
**22.3.3 Walking speed, ≥3 mo, <6 mo, Random**								
Bonnefoy 2003 [43], MET+PS vs PLA-S	10.2	39.5	53	6.5	38.1	49	12.6%	0.09 [-0.29, 0.48]
Chin A Paw 2001 [45], MET+PS vs PLA-S	0.06	0.05	42	0.01	0.001	37	11.6%	1.36 [0.86, 1.85]
Dirks 2017 [48], RET+PS vs RET+PLA-S	0	2.9	17	0.1	1.6	17	9.9%	-0.04 [-0.71, 0.63]
Franzke 2015 [50], RET+PS vs CG	42	44.7	27	11.2	23.9	45	11.5%	0.92 [0.42, 1.42]
Kim 2015 [54], MET+PS vs CG	0.1	0.08	33	0.007	0.08	98	12.3%	1.16 [0.74, 1.58]
Oesen 2015 [56], RET+PS vs CG	0	0.4	28	0.08	0.33	62	12.0%	-0.22 [-0.67, 0.22]
Rosendahl 2006 [57], MET+PS vs CG	0.02	0.12	42	-0.01	0.12	125	12.9%	0.25 [-0.10, 0.60]
Tieland 2012 [58], RET+PS vs RET+PLA-S	0	0.382	31	-0.2	0.354	31	11.5%	0.54 [0.03, 1.04]
Trabal 2015 [59], MET+PS vs MET+PLA-S	0.99	1.69	7	0.32	1.9	4	5.7%	0.35 [-0.89, 1.59]
**Subtotal (95% CI)**			**280**			**468**	**100.0%**	**0.50 [0.12, 0.88]**
Heterogeneity: Tau² = 0.26; Chi² = 42.47, df = 8 (P < 0.00001); I² = 81%								
Test for overall effect: Z = 2.59 (P = 0.010)								
**22.3.4 Walking speed, <3 mo; Fixed**								
Fiatarone 1994 [49], RET+PS vs CG	14.9	26.7	25	2.03	26.7	75	33.1%	0.48 [0.02, 0.94]
Niccoli 2017 [55], MET+PS vs MET+PLA-S	0.2	0.04	22	0.18	0.03	25	20.3%	0.56 [-0.02, 1.15]
Trabal 2015 [59], MET+PS vs MET+PLA-S	0.62	1.56	12	0.24	1.56	12	10.8%	0.24 [-0.57, 1.04]
Zak 2009 [61], MET+PS vs MET+PLA-S	36.4	55.1	19	35.7	58.9	21	18.0%	0.01 [-0.61, 0.63]
Zak 2009 [61], RET+PS vs RET+PLA-S	20.7	58.8	19	2.35	55.2	21	17.8%	0.32 [-0.31, 0.94]
**Subtotal (95% CI)**			**97**			**154**	**100.0%**	**0.36 [0.09, 0.62]**
Heterogeneity: Chi² = 2.03, df = 4 (P = 0.73); I² = 0%								
Test for overall effect: Z = 2.65 (P = 0.008)								

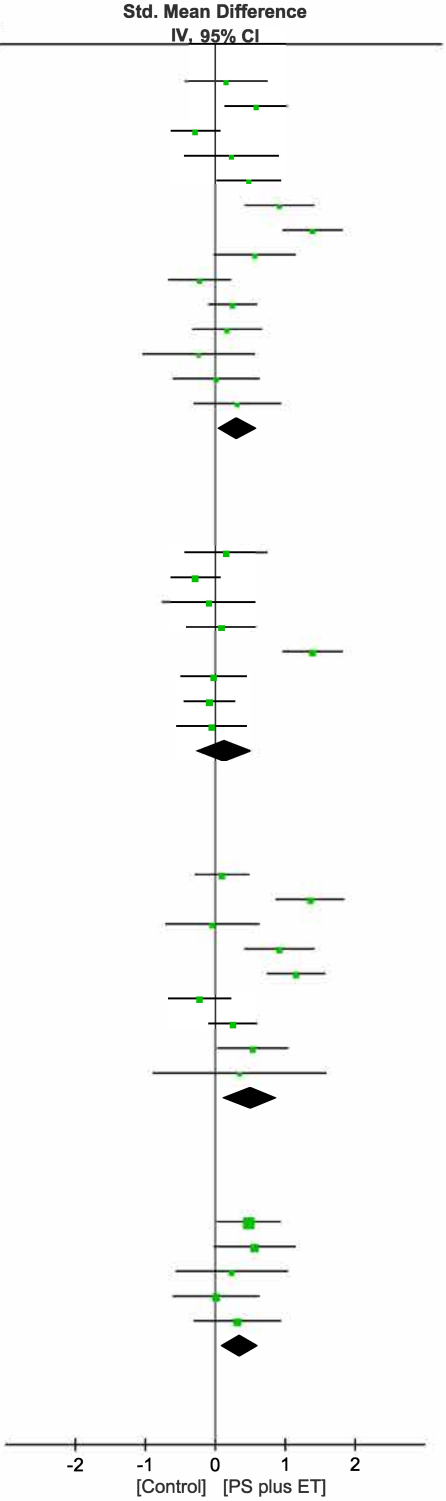

Figure S3. Forest plot summarizing effects of protein supplement (PS) plus exercise training (ET) on walk capability at an overall duration and each follow-up time point. The horizontal line links the lower and upper limits of the 95% CI of this effect. The combined effects are plotted using black diamonds. 95% CI = 95% confidence interval; Fixed = fixed-effects model; Random = random-effects model; Std. = standard; IV = inverse variance; CG = control group; Con = control; MET = multicomponent exercise training; PLA-S, placebo supplement; RET = resistance exercise training.

Study or Subgroup	PS plus ET Mean	PS plus ET SD	PS plus ET Total	Control Mean	Control SD	Control Total	Weight	Std. Mean Difference IV, Fixed, 95% CI
**22.4.1 Overall follow-up duration; Fixed**								
Beck 2010 [42], RET+PS vs RET	0.1	1.03	62	-0.7	1.33	59	83.8%	0.67 [0.30, 1.04]
Trabal 2015 [59], MET+PS vs MET+PLA-S	7.5	11.8	12	1	1.57	12	16.2%	0.75 [-0.09, 1.58]
**Subtotal (95% CI)**			**74**			**71**	**100.0%**	**0.68 [0.35, 1.02]**
Heterogeneity: Chi² = 0.03, df = 1 (P = 0.87); I² = 0%								
Test for overall effect: Z = 3.99 (P < 0.0001)								
**22.4.2 Exhaustion, ≥6 mo, <12 mo**								
Beck 2010 [42], RET+PS vs RET	0.5	1.71	62	0.25	1.83	59	100.0%	0.14 [-0.22, 0.50]
**Subtotal (95% CI)**			**62**			**59**	**100.0%**	**0.14 [-0.22, 0.50]**
Heterogeneity: Not applicable								
Test for overall effect: Z = 0.77 (P = 0.44)								
**22.4.3 Exhaustion, ≥3 mo, <6 mo; Fixed**								
Beck 2010 [42], RET+PS vs RET	0.1	1.03	62	-0.7	1.33	59	92.2%	0.67 [0.30, 1.04]
Trabal 2015 [59], MET+PS vs MET+PLA-S	0.5	0.5406	7	0	1.2	4	7.8%	0.56 [-0.71, 1.82]
**Subtotal (95% CI)**			**69**			**63**	**100.0%**	**0.66 [0.31, 1.01]**
Heterogeneity: Chi² = 0.03, df = 1 (P = 0.87); I² = 0%								
Test for overall effect: Z = 3.68 (P = 0.0002)								
**22.4.4 Exhaustion, <3 mo**								
Trabal 2015 [59], MET+PS vs MET+PLA-S	7.5	11.8	12	1	1.57	12	100.0%	0.75 [-0.09, 1.58]
**Subtotal (95% CI)**			**12**			**12**	**100.0%**	**0.75 [-0.09, 1.58]**
Heterogeneity: Not applicable								
Test for overall effect: Z = 1.75 (P = 0.08)								

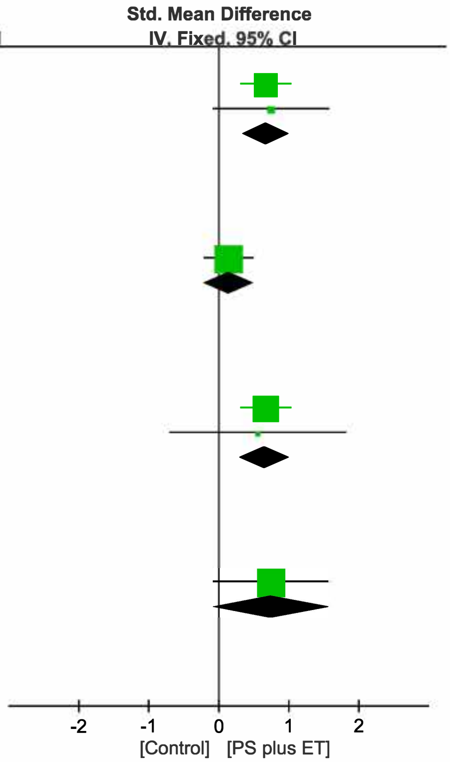

Figure S4. Forest plot summarizing effects of protein supplement (PS) plus exercise training (ET) on exhaustion at an overall duration and each follow-up time point. The horizontal line links the lower and upper limits of the 95% CI of this effect. The combined effects are plotted using black diamonds. 95% CI = 95% confidence interval; Fixed = fixed-effects model; Random = random-effects model; Std. = standard; IV = inverse variance; CG = control group; Con = control; MET = multicomponent exercise training; PLA-S, placebo supplement; RET = resistance exercise training.

Study or Subgroup	PS plus ET Mean	PS plus ET SD	PS plus ET Total	Control Mean	Control SD	Control Total	Weight	Std. Mean Difference IV, Random, 95% CI
**22.5.1 Overall follow-up duration; Random**								
Corcoran 2017 [46], MET+PS vs Con	-39	158	67	10	582	54	18.5%	-0.12 [-0.48, 0.24]
Fiatarone 1994 [49], RET+PS vs CG	553	3,487	25	756	3,497	75	16.9%	-0.06 [-0.51, 0.40]
Ikeda 2016 [52], MET+PS vs MET+PLA-S, Tr1	0.3	15.4	27	0.7	27.6	25	15.3%	-0.02 [-0.56, 0.53]
Ikeda 2016 [52], MET+PS vs MET+PLA-S, Tr2	-0.3	23.7	25	2	32.3	27	15.3%	-0.08 [-0.62, 0.46]
Kim 2015 [54], MET+PS vs CG	36.4	26.3	33	9.2	26.3	98	17.5%	1.03 [0.61, 1.44]
Oesen 2015 [56], RET+PS vs CG	-293	862	25	-469	1,179	57	16.6%	0.16 [-0.31, 0.63]
**Subtotal (95% CI)**			**202**			**336**	**100.0%**	**0.16 [-0.22, 0.54]**
Heterogeneity: Tau² = 0.17; Chi² = 21.24, df = 5 (P = 0.0007); I² = 76%								
Test for overall effect: Z = 0.82 (P = 0.41)								
**22.5.2 Physical activity, ≥6 mo, <12 mo; Random**								
Corcoran 2017 [46], MET+PS vs Con	-39	158	67	10	582	54	34.4%	-0.12 [-0.48, 0.24]
Kim 2015 [54], MET+PS vs CG	36.4	26.3	33	9.2	26.3	98	33.4%	1.03 [0.61, 1.44]
Oesen 2015 [56], RET+PS vs CG	-293	862	25	-469	1,179	57	32.3%	0.16 [-0.31, 0.63]
**Subtotal (95% CI)**			**125**			**209**	**100.0%**	**0.35 [-0.35, 1.06]**
Heterogeneity: Tau² = 0.34; Chi² = 17.45, df = 2 (P = 0.0002); I² = 89%								
Test for overall effect: Z = 0.98 (P = 0.33)								
**22.5.3 Physical activity, ≥3 mo, <6 mo; Fixed**								
Ikeda 2016 [52], MET+PS vs MET+PLA-S, Tr1	0.3	15.4	27	0.7	27.6	25	25.7%	-0.02 [-0.56, 0.53]
Ikeda 2016 [52], MET+PS vs MET+PLA-S, Tr2	-0.3	23.7	25	2	32.3	27	25.7%	-0.08 [-0.62, 0.46]
Kim 2015 [54], MET+PS vs CG	54.5	44	33	42.8	39.5	98	48.6%	0.29 [-0.11, 0.68]
**Subtotal (95% CI)**			**85**			**150**	**100.0%**	**0.11 [-0.16, 0.39]**
Heterogeneity: Chi² = 1.44, df = 2 (P = 0.49); I² = 0%								
Test for overall effect: Z = 0.81 (P = 0.42)								
**22.5.4 Physical activity, <3 mo**								
Fiatarone 1994 [49], RET+PS vs CG	553	3,487	25	756	3,497	75	100.0%	-0.06 [-0.51, 0.40]
**Subtotal (95% CI)**			**25**			**75**	**100.0%**	**-0.06 [-0.51, 0.40]**
Heterogeneity: Not applicable								
Test for overall effect: Z = 0.25 (P = 0.80)								

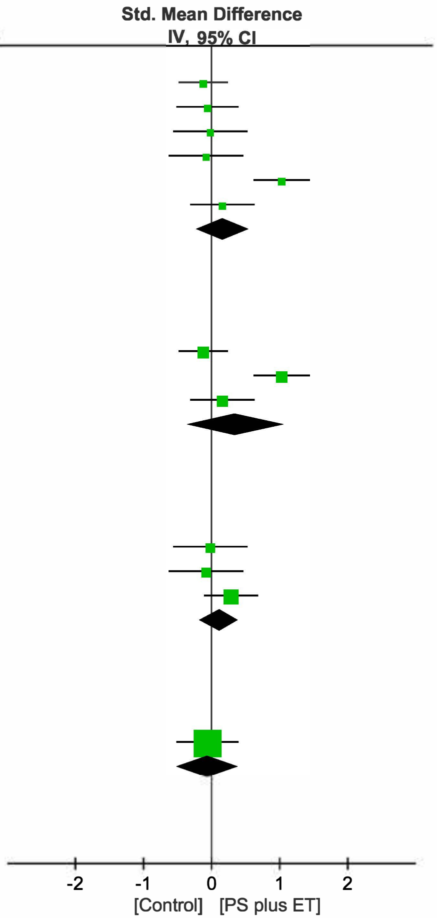

Figure S5. Forest plot summarizing effects of protein supplement (PS) plus exercise training (ET) on physical activity at an overall duration and each follow-up time point. The horizontal line links the lower and upper limits of the 95% CI of this effect. The combined effects are plotted using black diamonds. 95% CI = 95% confidence interval; Fixed = fixed-effects model; Random = random-effects model; Std. = standard; IV = inverse variance; CG = control group; Con = control; MET = multicomponent exercise training; PLA-S, placebo supplement; RET = resistance exercise training; Tr = treatment session.

Study or Subgroup	PS plus ET Mean	PS plus ET SD	PS plus ET Total	Control Mean	Control SD	Control Total	Weight	Std. Mean Difference IV, Fixed, 95% CI
**22.6.1 Overall follow-up duration**								
Kim 2015 [54], MET+PS vs CG	1.3	1.1	33	0.57	1.2	87	100.0%	0.62 [0.21, 1.03]
**Subtotal (95% CI)**			**33**			**87**	**100.0%**	**0.62 [0.21, 1.03]**
Heterogeneity: Not applicable								
Test for overall effect: Z = 2.96 (P = 0.003)								
**22.6.2 Global frailty score, ≥6 mo, <12 mo**								
Kim 2015 [54], MET+PS vs CG	1.3	1.1	33	0.57	1.2	87	100.0%	0.62 [0.21, 1.03]
**Subtotal (95% CI)**			**33**			**87**	**100.0%**	**0.62 [0.21, 1.03]**
Heterogeneity: Not applicable								
Test for overall effect: Z = 2.96 (P = 0.003)								
**22.6.3 Global frailty score, ≥3 mo, <6 mo**								
Kim 2015 [54], MET+PS vs CG	1.3	1.1	33	0.76	1.2	98	100.0%	0.46 [0.06, 0.85]
**Subtotal (95% CI)**			**33**			**98**	**100.0%**	**0.46 [0.06, 0.85]**
Heterogeneity: Not applicable								
Test for overall effect: Z = 2.25 (P = 0.02)								
**22.6.4 Global frailty score, <3 mo**								
**Subtotal (95% CI)**			**0**			**0**		**Not estimable**
Heterogeneity: Not applicable								
Test for overall effect: Not applicable								

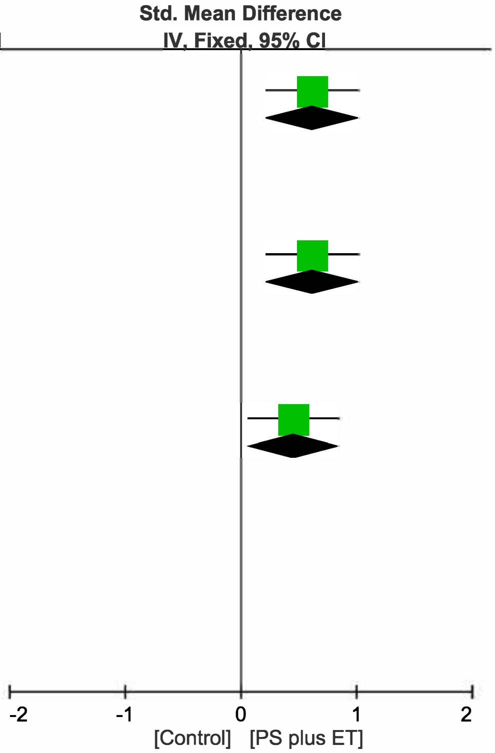

Figure S6. Forest plot summarizing effects of protein supplement (PS) plus exercise training (ET) on global frailty score at an overall duration and each follow-up time point. The horizontal line links the lower and upper limits of the 95% CI of this effect. The combined effects are plotted using black diamonds. 95% CI = 95% confidence interval; Fixed = fixed-effects model; Std. = standard; IV = inverse variance; CG = control group; MET = multicomponent exercise training; PLA-S, placebo supplement; RET = resistance exercise training.

Study or Subgroup	PS plus ET Mean	PS plus ET SD	PS plus ET Total	Control Mean	Control SD	Control Total	Weight	Std. Mean Difference IV, Fixed, 95% CI
**22.7.1 Overall follow-up duration; Fixed**								
Bonnefoy 2003 [43], MET+PS vs PLA-S	1.4	3.5	20	-0.82	3.7	20	8.8%	0.60 [-0.03, 1.24]
Carlsson 2011 [44], MET+PS vs CG	0	1.84	36	-0.2	2.48	113	25.2%	0.08 [-0.29, 0.46]
Dirks 2017 [48], RET+PS vs RET+PLA-S	1.4	2.3	17	-0.1	1.97	17	7.4%	0.68 [-0.01, 1.38]
Fiatarone 1994 [49], RET+PS vs CG	1.5	3.91	25	0.5	4.22	75	17.2%	0.24 [-0.21, 0.69]
Imaoka 2016 [53], MET+PS vs CG	0.4	0.91	23	-0.2	0.59	68	14.8%	0.87 [0.38, 1.36]
Tieland 2012 [58], RET+PS vs RET+PLA-S	1.2	1.3	28	-0.1	1.3	28	11.4%	0.99 [0.43, 1.54]
Yamada 2015 [60], RET+PS vs CG	0.2	0.3	31	-0.1	0.48	40	15.1%	0.72 [0.24, 1.21]
**Subtotal (95% CI)**			**180**			**361**	**100.0%**	**0.52 [0.33, 0.71]**
Heterogeneity: Chi² = 12.23, df = 6 (P = 0.06); I² = 51%								
Test for overall effect: Z = 5.38 (P < 0.00001)								
**22.7.2 Lean body mass, ≥6 mo, <12 mo; Fixed**								
Bonnefoy 2003 [43], MET+PS vs PLA-S	0.8	4.4	20	-1.1	4.4	20	13.2%	0.42 [-0.20, 1.05]
Carlsson 2011 [44], MET+PS vs CG	-0.1	2.37	37	-0.3	3.1	102	36.7%	0.07 [-0.31, 0.44]
Dirks 2017 [48], RET+PS vs RET+PLA-S	1.4	2.3	17	-0.1	1.97	17	10.8%	0.68 [-0.01, 1.38]
Tieland 2012 [58], RET+PS vs RET+PLA-S	1.25	2.7	28	-0.4	1.83	28	17.7%	0.71 [0.16, 1.25]
Yamada 2015 [60], RET+PS vs CG	0.2	0.3	31	-0.15	0.48	40	21.6%	0.84 [0.35, 1.33]
**Subtotal (95% CI)**			**133**			**207**	**100.0%**	**0.46 [0.23, 0.69]**
Heterogeneity: Chi² = 7.70, df = 4 (P = 0.10); I² = 48%								
Test for overall effect: Z = 3.97 (P < 0.0001)								
**22.7.3 Lean body mass, ≥3 mo, <6 mo; Random**								
Bonnefoy 2003 [43], MET+PS vs PLA-S	1.4	3.5	20	-0.82	3.7	20	17.9%	0.60 [-0.03, 1.24]
Carlsson 2011 [44], MET+PS vs CG	0	1.84	36	-0.2	2.48	113	24.4%	0.08 [-0.29, 0.46]
Dirks 2017 [48], RET+PS vs RET+PLA-S	1.3	2.3	17	0.1	1.97	17	16.7%	0.55 [-0.14, 1.23]
Imaoka 2016 [53], MET+PS vs CG	0.44	0.91	23	-0.29	0.59	68	21.2%	1.06 [0.56, 1.56]
Tieland 2012 [58], RET+PS vs RET+PLA-S	1.2	1.3	28	-0.1	1.3	28	19.8%	0.99 [0.43, 1.54]
**Subtotal (95% CI)**			**124**			**246**	**100.0%**	**0.64 [0.22, 1.06]**
Heterogeneity: Tau² = 0.15; Chi² = 12.28, df = 4 (P = 0.02); I² = 67%								
Test for overall effect: Z = 2.99 (P = 0.003)								
**22.7.4 Lean body mass, <3 mo**								
Fiatarone 1994 [49], RET+PS vs CG	1.5	3.91	25	0.5	4.22	75	100.0%	0.24 [-0.21, 0.69]
**Subtotal (95% CI)**			**25**			**75**	**100.0%**	**0.24 [-0.21, 0.69]**
Heterogeneity: Not applicable								
Test for overall effect: Z = 1.03 (P = 0.30)								

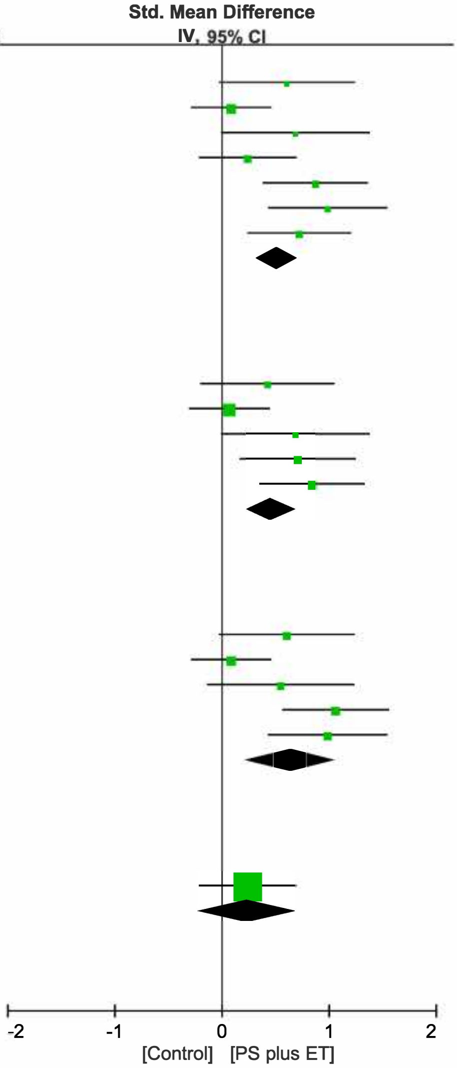

Figure S7. Forest plot summarizing effects of protein supplement (PS) plus exercise training (ET) on lean body mass at an overall duration and each follow-up time point. The horizontal line links the lower and upper limits of the 95% CI of this effect. The combined effects are plotted using black diamonds. 95% CI = 95% confidence interval; Fixed = fixed-effects model; Std. = standard; IV = inverse variance; CG = control group; Con = control; MET = multicomponent exercise training; PLA-S, placebo supplement; RET = resistance exercise training.

Study or Subgroup	PS plus ET Mean	PS plus ET SD	PS plus ET Total	Control Mean	Control SD	Control Total	Weight	Std. Mean Difference IV, Fixed, 95% CI
**22.8.1 Overall follow-up duration; Fixed**								
Dirks 2017 [48], RET+PS vs RET+PLA-S	1	1.22	17	0	0.95	17	17.0%	0.89 [0.18, 1.60]
Kim 2015 [54], MET+PS vs CG	0.31	0.33	33	0.12	0.36	98	53.5%	0.54 [0.14, 0.94]
Tieland 2012 [58], RET+PS vs RET+PLA-S	0.95	1.43	28	-0.2	1.91	28	29.4%	0.67 [0.13, 1.21]
**Subtotal (95% CI)**			**78**			**143**	**100.0%**	**0.64 [0.34, 0.93]**
Heterogeneity: Chi² = 0.76, df = 2 (P = 0.68); I² = 0%								
Test for overall effect: Z = 4.26 (P < 0.0001)								
**22.8.2 Appendicular lean mass, ≥6 mo, <12 mo; Fixed**								
Dirks 2017 [48], RET+PS vs RET+PLA-S	1	1.22	17	0	0.95	17	16.8%	0.89 [0.18, 1.60]
Kim 2015 [54], MET+PS vs CG	0.44	0.37	33	0.35	0.4	98	54.1%	0.23 [-0.17, 0.62]
Tieland 2012 [58], RET+PS vs RET+PLA-S	0.95	1.43	28	-0.2	1.91	28	29.1%	0.67 [0.13, 1.21]
**Subtotal (95% CI)**			**78**			**143**	**100.0%**	**0.47 [0.18, 0.76]**
Heterogeneity: Chi² = 3.35, df = 2 (P = 0.19); I² = 40%								
Test for overall effect: Z = 3.16 (P = 0.002)								
**22.8.3 Appendicular lean mass, ≥3 mo, <6 mo; Fixed**								
Dirks 2017 [48], RET+PS vs RET+PLA-S	0.4	1.07	17	0.1	0.95	17	18.1%	0.29 [-0.39, 0.97]
Kim 2015 [54], MET+PS vs CG	0.31	0.33	33	0.12	0.36	98	51.8%	0.54 [0.14, 0.94]
Tieland 2012 [58], RET+PS vs RET+PLA-S	0.4	0.64	28	0	2.61	28	30.0%	0.21 [-0.32, 0.73]
**Subtotal (95% CI)**			**78**			**143**	**100.0%**	**0.39 [0.10, 0.68]**
Heterogeneity: Chi² = 1.06, df = 2 (P = 0.59); I² = 0%								
Test for overall effect: Z = 2.67 (P = 0.008)								
**22.8.4 Appendicular lean mass, <3 mo**								
**Subtotal (95% CI)**			**0**			**0**		**Not estimable**
Heterogeneity: Not applicable								
Test for overall effect: Not applicable								

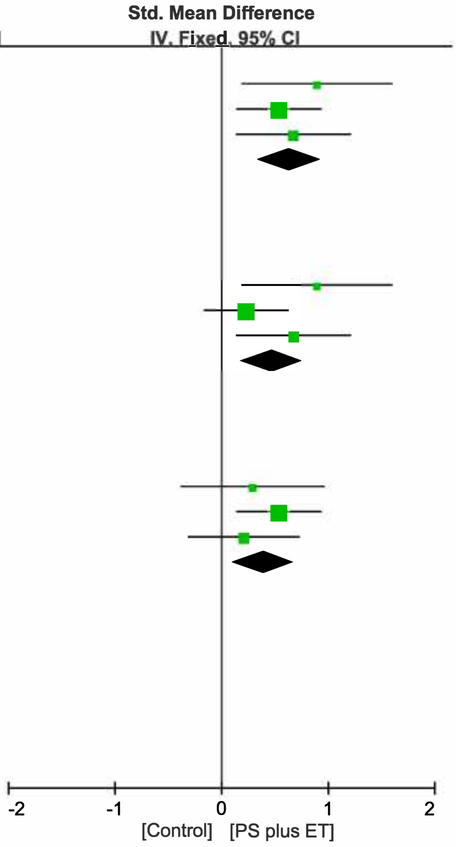

Figure S8. Forest plot summarizing effects of protein supplement (PS) plus exercise training (ET) on appendicular lean mass at an overall duration and each follow-up time point. The horizontal line links the lower and upper limits of the 95% CI of this effect. The combined effects are plotted using black diamonds. 95% CI = 95% confidence interval; Fixed = fixed-effects model; Std. = standard; IV = inverse variance; CG = control group; CogT = cognition training; Con = control; MET = multicomponent exercise training; PLA-S, placebo supplement; RET = resistance exercise training.

Study or Subgroup	PS plus ET Mean	PS plus ET SD	PS plus ET Total	Control Mean	Control SD	Control Total	Weight	Std. Mean Difference IV, Fixed, 95% CI
**22.9.1 Overall follow-up duration; Fixed**								
Dirks 2017 [48], RET+PS vs RET+PLA-S	0	2.37	17	-0.1	1.89	17	37.8%	0.05 [-0.63, 0.72]
Tieland 2012 [58], RET+PS vs RET+PLA-S	0.2	1.91	28	0	1.83	28	62.2%	0.11 [-0.42, 0.63]
**Subtotal (95% CI)**			**45**			**45**	**100.0%**	**0.08 [-0.33, 0.50]**
Heterogeneity: Chi² = 0.02, df = 1 (P = 0.89); I² = 0%								
Test for overall effect: Z = 0.39 (P = 0.69)								
**22.9.2 Fat mass, ≥6 mo, <12 mo; Fixed**								
Dirks 2017 [48], RET+PS vs RET+PLA-S	0.9	2.38	17	-0.5	1.99	17	37.8%	0.62 [-0.07, 1.31]
Tieland 2012 [58], RET+PS vs RET+PLA-S	0.7	1.91	28	-0.55	1.85	28	62.2%	0.66 [0.12, 1.19]
**Subtotal (95% CI)**			**45**			**45**	**100.0%**	**0.64 [0.22, 1.07]**
Heterogeneity: Chi² = 0.01, df = 1 (P = 0.94); I² = 0%								
Test for overall effect: Z = 2.97 (P = 0.003)								
**22.9.3 Fat mass, ≥3 mo, <6 mo; Fixed**								
Dirks 2017 [48], RET+PS vs RET+PLA-S	0	2.37	17	-0.1	1.89	17	37.8%	0.05 [-0.63, 0.72]
Tieland 2012 [58], RET+PS vs RET+PLA-S	0.2	1.91	28	0	1.83	28	62.2%	0.11 [-0.42, 0.63]
**Subtotal (95% CI)**			**45**			**45**	**100.0%**	**0.08 [-0.33, 0.50]**
Heterogeneity: Chi² = 0.02, df = 1 (P = 0.89); I² = 0%								
Test for overall effect: Z = 0.39 (P = 0.69)								
**22.9.4 Fat mass, <3 mo**								
**Subtotal (95% CI)**			**0**			**0**		**Not estimable**
Heterogeneity: Not applicable								
Test for overall effect: Not applicable								

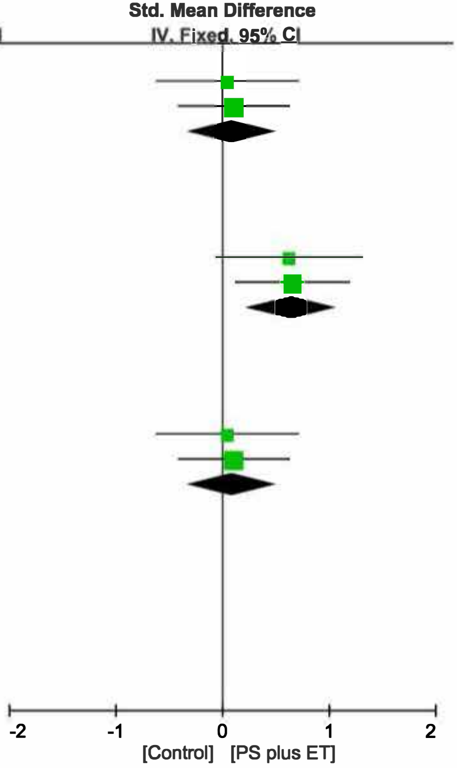

Figure S9. Forest plot summarizing effects of protein supplement (PS) plus exercise training (ET) on fat mass at an overall duration and each follow-up time point. The horizontal line links the lower and upper limits of the 95% CI of this effect. The combined effects are plotted using black diamonds. 95% CI = 95% confidence interval; Fixed = fixed-effects model; Std. = standard; IV = inverse variance; CG = control group; Con = control; MET = multicomponent exercise training; PLA-S, placebo supplement; RET = resistance exercise training.

Study or Subgroup	PS plus ET Mean	PS plus ET SD	PS plus ET Total	Control Mean	Control SD	Control Total	Weight	Std. Mean Difference IV, Fixed, 95% CI
**22.10.1 Overall follow-up duration; Fixed**								
Bonnefoy 2003 [43], MET+PS vs PLA-S	30.12	66	20	-12.12	30.8	20	4.4%	0.80 [0.16, 1.45]
Chin A Paw 2001 [45], MET+PS vs PLA-S	2	5.3	42	0.7	4.7	37	9.4%	0.26 [-0.19, 0.70]
Dirks 2017 [48], RET+PS vs RET+PLA-S	51	34.28	17	49	7.33	17	4.1%	0.08 [-0.59, 0.75]
Fiatarone 1994 [49], RET+PS vs CG	14.9	9	25	3.58	10.7	75	8.1%	1.09 [0.61, 1.57]
Hofmann 2016 [51], RET+PS vs CG	1.44	7.94	24	-0.56	7.41	52	7.8%	0.26 [-0.22, 0.75]
Ikeda 2016 [52], MET+PS vs MET+PLA-S, Tr1	0.03	0.13	27	-0.01	0.15	25	6.2%	0.28 [-0.27, 0.83]
Ikeda 2016 [52], MET+PS vs MET+PLA-S, Tr2	0.07	0.11	25	-0.01	0.13	27	5.9%	0.65 [0.09, 1.21]
Kim 2015 [54], MET+PS vs CG	0.38	3.41	33	0.3	3.38	98	11.8%	0.02 [-0.37, 0.42]
Niccoli 2017 [55], MET+PS vs MET+PLA-S	5.89	3.76	22	3.42	0.84	25	5.0%	0.92 [0.31, 1.52]
Oesen 2015 [56], RET+PS vs CG	0.11	0.18	25	0.03	0.17	57	8.1%	0.46 [-0.02, 0.93]
Rosendahl 2006 [57], MET+PS vs CG	11.6	28.8	28	4.6	19.7	69	9.5%	0.31 [-0.13, 0.75]
Tieland 2012 [58], RET+PS vs RET+PLA-S	32	25.9	31	31	25.5	31	7.4%	0.04 [-0.46, 0.54]
Trabal 2015 [59], MET+PS vs MET+PLA-S	9.97	8.68	12	5.03	8.69	12	2.8%	0.55 [-0.27, 1.37]
Zak 2009 [61], MET+PS vs MET+PLA-S	1.7	8.9	19	1.4	9	21	4.8%	0.03 [-0.59, 0.65]
Zak 2009 [61], RET+PS vs RET+PLA-S	5.6	9.6	19	3.8	9.7	21	4.8%	0.18 [-0.44, 0.80]
**Subtotal (95% CI)**			**369**			**587**	**100.0%**	**0.37 [0.23, 0.51]**
Heterogeneity: Chi^2 = 22.39, df = 14 (P = 0.07); I^2 = 37%								
Test for overall effect: Z = 5.35 (P < 0.00001)								
**22.10.2 Leg strength, ≥6 mo, <12 mo; Fixed**								
Bonnefoy 2003 [43], MET+PS vs PLA-S	56.87	92.4	20	18.63	79.2	20	8.9%	0.44 [-0.19, 1.06]
Dirks 2017 [48], RET+PS vs RET+PLA-S	51	34.28	17	49	7.33	17	7.8%	0.08 [-0.59, 0.75]
Hofmann 2016 [51], RET+PS vs CG	3.24	8.29	21	2.27	9.3	49	13.4%	0.11 [-0.41, 0.62]
Kim 2015 [54], MET+PS vs CG	-0.92	3.11	33	0.39	3.31	98	22.2%	-0.40 [-0.80, -0.00]
Oesen 2015 [56], RET+PS vs CG	0.11	0.18	25	0.03	0.17	57	15.5%	0.46 [-0.02, 0.93]
Rosendahl 2006 [57], MET+PS vs CG	11.6	28.8	28	4.6	19.7	69	18.0%	0.31 [-0.13, 0.75]
Tieland 2012 [58], RET+PS vs RET+PLA-S	45	25.86	31	46	25.99	31	14.2%	-0.04 [-0.54, 0.46]
**Subtotal (95% CI)**			**175**			**341**	**100.0%**	**0.09 [-0.10, 0.28]**
Heterogeneity: Chi^2 = 10.47, df = 6 (P = 0.11); I^2 = 43%								
Test for overall effect: Z = 0.96 (P = 0.34)								
**22.10.3 Leg strength, ≥3 mo, <6 mo; Fixed**								
Bonnefoy 2003 [43], MET+PS vs PLA-S	30.12	66	20	-12.12	30.8	20	5.7%	0.80 [0.16, 1.45]
Chin A Paw 2001 [45], MET+PS vs PLA-S	2	5.3	42	0.7	4.7	37	12.0%	0.26 [-0.19, 0.70]
Dirks 2017 [48], RET+PS vs RET+PLA-S	31	24.6	17	39	6.96	17	5.1%	-0.43 [-1.11, 0.25]
Hofmann 2016 [51], RET+PS vs CG	1.44	7.94	24	-0.56	7.41	52	10.1%	0.26 [-0.22, 0.75]
Ikeda 2016 [52], MET+PS vs MET+PLA-S, Tr1	0.03	0.13	27	-0.01	0.15	25	7.9%	0.28 [-0.27, 0.83]
Ikeda 2016 [52], MET+PS vs MET+PLA-S, Tr2	0.07	0.11	25	-0.01	0.13	27	7.6%	0.65 [0.09, 1.21]
Kim 2015 [54], MET+PS vs CG	0.38	3.41	33	0.3	3.38	98	15.2%	0.02 [-0.37, 0.42]
Oesen 2015 [56], RET+PS vs CG	0.15	0.2	28	0.13	0.2	62	11.9%	0.10 [-0.35, 0.55]
Rosendahl 2006 [57], MET+PS vs CG	11.3	21.2	31	8.7	21	72	13.4%	0.12 [-0.30, 0.54]
Tieland 2012 [58], RET+PS vs RET+PLA-S	32	25.9	31	31	25.5	31	9.6%	0.04 [-0.46, 0.54]
Trabal 2015 [59], MET+PS vs MET+PLA-S	12.5	10.7	7	6.01	13.5	4	1.5%	0.51 [-0.75, 1.76]
**Subtotal (95% CI)**			**285**			**445**	**100.0%**	**0.20 [0.04, 0.35]**
Heterogeneity: Chi^2 = 11.10, df = 10 (P = 0.35); I^2 = 10%								
Test for overall effect: Z = 2.49 (P = 0.01)								
**22.10.4 Leg strength, <3 mo; Random**								
Fiatarone 1994 [49], RET+PS vs CG	14.9	9	25	3.58	10.7	75	24.0%	1.09 [0.61, 1.57]
Niccoli 2017 [55], MET+PS vs MET+PLA-S	5.89	3.76	22	3.42	0.84	25	20.5%	0.92 [0.31, 1.52]
Trabal 2015 [59], MET+PS vs MET+PLA-S	9.97	8.68	12	5.03	8.69	12	15.4%	0.55 [-0.27, 1.37]
Zak 2009 [61], MET+PS vs MET+PLA-S	1.7	8.9	19	1.4	9	21	20.1%	0.03 [-0.59, 0.65]
Zak 2009 [61], RET+PS vs RET+PLA-S	5.6	9.6	19	3.8	9.7	21	20.0%	0.18 [-0.44, 0.80]
**Subtotal (95% CI)**			**97**			**154**	**100.0%**	**0.58 [0.14, 1.01]**
Heterogeneity: Tau^2 = 0.15; Chi^2 = 10.01, df = 4 (P = 0.04); I^2 = 60%								
Test for overall effect: Z = 2.60 (P = 0.009)								

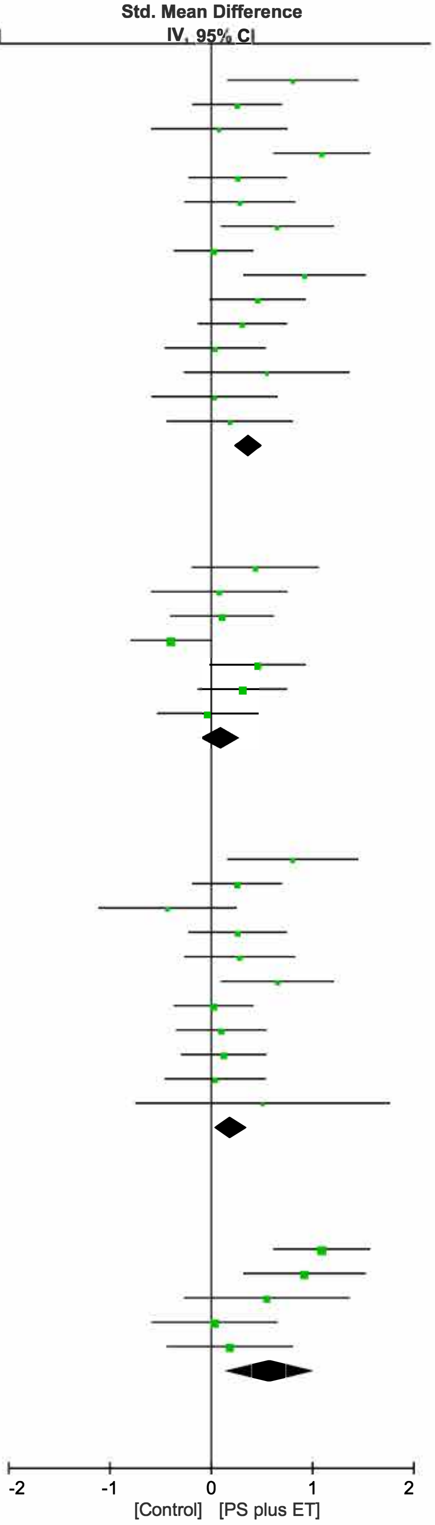

Figure S10. Forest plot summarizing effects of protein supplement (PS) plus exercise training (ET) on leg strength at an overall duration and each follow-up time point. The horizontal line links the lower and upper limits of the 95% CI of this effect. The combined effects are plotted using black diamonds. 95% CI = 95% confidence interval; Fixed = fixed-effects model; Std. = standard; IV = inverse variance; CG = control group; CogT = cognition training; Con = control; MET = multicomponent exercise training; PLA-S, placebo supplement; RET = resistance exercise training; Tr = treatment session.

Study or Subgroup	PS plus ET Mean	PS plus ET SD	PS plus ET Total	Control Mean	Control SD	Control Total	Weight	Std. Mean Difference IV, Fixed, 95% CI
**22.11.1 Overall follow-up duration; Fixed**								
Beck 2016 [41], MET+PS vs Con, free living	0.9	1.97	46	1.1	2.48	18	12.1%	-0.09 [-0.64, 0.45]
Beck 2016 [41], MET+PS vs Con, nursing home	-12.1	3.97	9	-11.9	3.75	22	6.0%	-0.05 [-0.83, 0.72]
Bonnefoy 2003 [43], MET+PS vs PLA-S	24.2	18.4	22	7.5	32.2	22	9.8%	0.63 [0.02, 1.23]
Chin A Paw 2001 [45], MET+PS vs PLA-S	3.2	3	42	1.7	3	37	17.9%	0.50 [0.05, 0.94]
Dirks 2017 [48], RET+PS vs RET+PLA-S	2.8	2.5	17	3.9	3.46	17	7.8%	-0.36 [-1.03, 0.32]
Franzke 2015 [50], RET+PS vs CG	3.1	4.7	22	1.1	9.1	48	14.1%	0.25 [-0.26, 0.75]
Oesen 2015 [56], RET+PS vs CG	3	3	25	1.1	2.7	57	15.5%	0.67 [0.19, 1.16]
Tieland 2012 [58], RET+PS vs RET+PLA-S	1.95	3.81	31	0.9	3.68	31	14.4%	0.28 [-0.22, 0.78]
Trabal 2015 [59], MET+PS vs MET+PLA-S	5.2	5	7	1.9	7	4	2.3%	0.53 [-0.73, 1.78]
**Subtotal (95% CI)**			**221**			**256**	**100.0%**	**0.30 [0.11, 0.49]**
Heterogeneity: Chi² = 10.69, df = 8 (P = 0.22); I² = 25%								
Test for overall effect: Z = 3.09 (P = 0.002)								
**22.11.2 Chair rise, ≥6 mo, <12 mo; Random**								
Bonnefoy 2003 [43], MET+PS vs PLA-S	24.2	18.4	22	7.5	32.2	22	19.1%	0.63 [0.02, 1.23]
Dirks 2017 [48], RET+PS vs RET+PLA-S	2.8	2.5	17	3.9	3.46	17	17.8%	-0.36 [-1.03, 0.32]
Franzke 2015 [50], RET+PS vs CG	3.1	4.7	22	1.1	9.1	48	20.9%	0.25 [-0.26, 0.75]
Oesen 2015 [56], RET+PS vs CG	3	3	25	1.1	2.7	57	21.4%	0.67 [0.19, 1.16]
Tieland 2012 [58], RET+PS vs RET+PLA-S	2.1	3.86	31	4.15	3.81	31	20.9%	-0.53 [-1.03, -0.02]
**Subtotal (95% CI)**			**117**			**175**	**100.0%**	**0.14 [-0.35, 0.63]**
Heterogeneity: Tau² = 0.23; Chi² = 16.03, df = 4 (P = 0.003); I² = 75%								
Test for overall effect: Z = 0.56 (P = 0.57)								
**22.11.3 Chair rise, ≥3 mo, <6 mo; Fixed**								
Chin A Paw 2001 [45], MET+PS vs PLA-S	3.2	3	42	1.7	3	37	23.5%	0.50 [0.05, 0.94]
Dirks 2017 [48], RET+PS vs RET+PLA-S	0.2	6.44	17	2.5	2.95	17	10.2%	-0.45 [-1.13, 0.23]
Franzke 2015 [50], RET+PS vs CG	1.3	1.7	27	0.8	2.8	45	20.7%	0.20 [-0.28, 0.68]
Oesen 2015 [56], RET+PS vs CG	1	3	28	0.5	2	62	23.7%	0.21 [-0.24, 0.66]
Tieland 2012 [58], RET+PS vs RET+PLA-S	1.95	3.81	31	0.9	3.68	31	18.9%	0.28 [-0.22, 0.78]
Trabal 2015 [59], MET+PS vs MET+PLA-S	5.2	5	7	1.9	7	4	3.0%	0.53 [-0.73, 1.78]
**Subtotal (95% CI)**			**152**			**196**	**100.0%**	**0.23 [0.01, 0.45]**
Heterogeneity: Chi² = 5.41, df = 5 (P = 0.37); I² = 8%								
Test for overall effect: Z = 2.08 (P = 0.04)								
**22.11.4 Chair rise, <3 mo; Fixed**								
Beck 2016 [41], MET+PS vs Con, free living	0.9	1.97	46	1.1	2.48	18	51.1%	-0.09 [-0.64, 0.45]
Beck 2016 [41], MET+PS vs Con, nursing home	-12.1	3.97	9	-11.9	3.75	22	25.2%	-0.05 [-0.83, 0.72]
Trabal 2015 [59], MET+PS vs MET+PLA-S	3.58	4.52	12	2.83	5.25	12	23.6%	0.15 [-0.65, 0.95]
**Subtotal (95% CI)**			**67**			**52**	**100.0%**	**-0.03 [-0.42, 0.36]**
Heterogeneity: Chi² = 0.24, df = 2 (P = 0.89); I² = 0%								
Test for overall effect: Z = 0.13 (P = 0.90)								

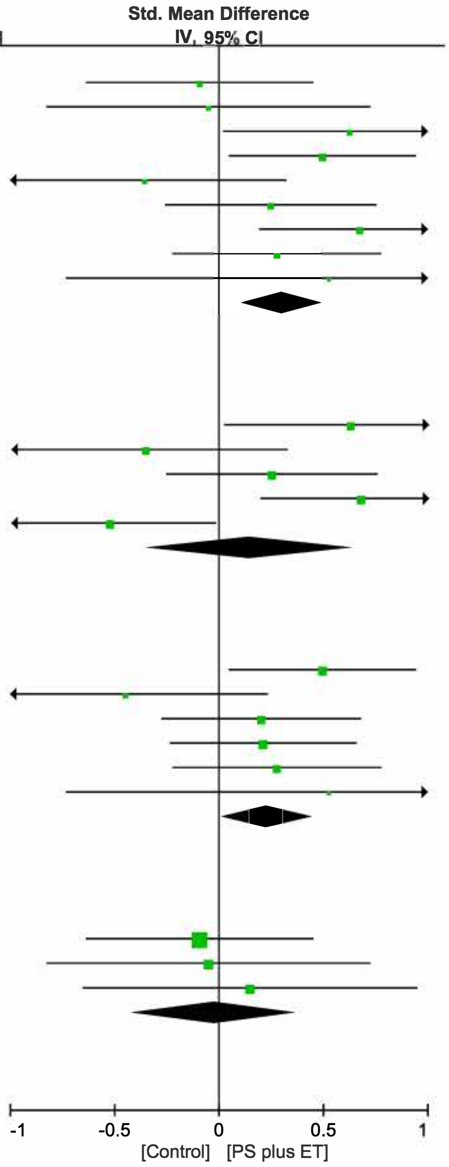

Figure S11. Forest plot summarizing effects of protein supplement (PS) plus exercise training (ET) on chair rise at an overall duration and each follow-up time point. The horizontal line links the lower and upper limits of the 95% CI of this effect. The combined effects are plotted using black diamonds. 95% CI = 95% confidence interval; Fixed = fixed-effects model; Random = random-effects model; Std. = standard; IV = inverse variance; CG = control group; Con = control; MET = multicomponent exercise training; PLA-S, placebo supplement; RET = resistance exercise training.

Study or Subgroup	PS plus ET Mean	PS plus ET SD	PS plus ET Total	Control Mean	Control SD	Control Total	Weight	Std. Mean Difference IV, Fixed, 95% CI
**22.12.1 Overall follow-up duration; Fixed**								
Ikeda 2016 [52], MET+PS vs MET+PLA-S, Tr1	0.5	8.52	27	1.6	9.68	25	20.3%	-0.12 [-0.66, 0.43]
Ikeda 2016 [52], MET+PS vs MET+PLA-S, Tr2	0.2	5.91	25	0.3	6.11	27	20.3%	-0.02 [-0.56, 0.53]
Kim 2015 [54], MET+PS vs CG	1.74	1	33	0.9	1.5	98	37.4%	0.60 [0.20, 1.00]
Niccoli 2017 [55], MET+PS vs MET+PLA-S	6.88	2.12	22	6.4	1.91	25	18.2%	0.23 [-0.34, 0.81]
Trabal 2015 [59], MET+PS vs MET+PLA-S	3.54	4.3	7	0.8	4.6	4	3.8%	0.57 [-0.69, 1.83]
**Subtotal (95% CI)**			**114**			**179**	**100.0%**	**0.26 [0.02, 0.51]**
Heterogeneity: Chi² = 5.84, df = 4 (P = 0.21); I² = 32%								
Test for overall effect: Z = 2.08 (P = 0.04)								
**22.12.2 TUG, ≥6 mo, <12 mo**								
Kim 2015 [54], MET+PS vs CG	2.7	1.15	33	2.7	1.98	98	100.0%	0.00 [-0.39, 0.39]
**Subtotal (95% CI)**			**33**			**98**	**100.0%**	**0.00 [-0.39, 0.39]**
Heterogeneity: Not applicable								
Test for overall effect: Z = 0.00 (P = 1.00)								
**22.12.3 TUG, ≥3 mo, <6 mo; Fixed**								
Ikeda 2016 [52], MET+PS vs MET+PLA-S, Tr1	0.5	8.52	27	1.6	9.68	25	24.8%	-0.12 [-0.66, 0.43]
Ikeda 2016 [52], MET+PS vs MET+PLA-S, Tr2	0.2	5.91	25	0.3	6.11	27	24.9%	-0.02 [-0.56, 0.53]
Kim 2015 [54], MET+PS vs CG	1.74	1	33	0.9	1.5	98	45.7%	0.60 [0.20, 1.00]
Trabal 2015 [59], MET+PS vs MET+PLA-S	3.54	4.3	7	0.8	4.6	4	4.6%	0.57 [-0.69, 1.83]
**Subtotal (95% CI)**			**92**			**154**	**100.0%**	**0.27 [-0.00, 0.54]**
Heterogeneity: Chi² = 5.83, df = 3 (P = 0.12); I² = 49%								
Test for overall effect: Z = 1.93 (P = 0.05)								
**22.12.4 TUG, <3 mo; Fixed**								
Niccoli 2017 [55], MET+PS vs MET+PLA-S	6.88	2.12	22	6.4	1.91	25	66.6%	0.23 [-0.34, 0.81]
Trabal 2015 [59], MET+PS vs MET+PLA-S	3.14	4.14	12	1.25	4.14	12	33.4%	0.44 [-0.37, 1.25]
**Subtotal (95% CI)**			**34**			**37**	**100.0%**	**0.30 [-0.17, 0.77]**
Heterogeneity: Chi² = 0.16, df = 1 (P = 0.68); I² = 0%								
Test for overall effect: Z = 1.27 (P = 0.20)								

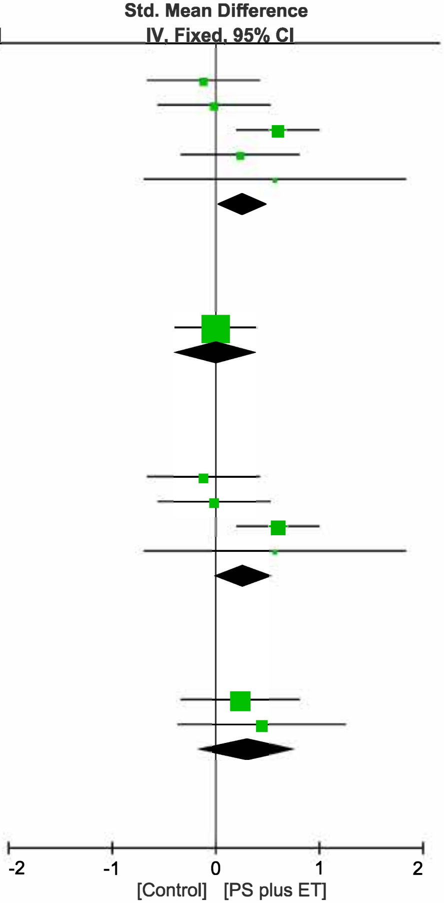

Figure S12. Forest plot summarizing effects of protein supplement (PS) plus exercise training (ET) on chair rise at an overall duration and each follow-up time point. The horizontal line links the lower and upper limits of the 95% CI of this effect. The combined effects are plotted using black diamonds. 95% CI = 95% confidence interval; Fixed = fixed-effects model; Random = random-effects model; Std. = standard; IV = inverse variance; CG = control group; Con = control; MET = multicomponent exercise training; PLA-S, placebo supplement; RET = resistance exercise training.

Study or Subgroup	PS plus ET Mean	PS plus ET SD	PS plus ET Total	Control Mean	Control SD	Control Total	Weight	Std. Mean Difference IV, Fixed, 95% CI
**22.13.1 Overall follow-up duration; Fixed**								
Corcoran 2017 [46], MET+PS vs Con	0.6	2.9	67	0.4	3.4	54	56.8%	0.06 [-0.30, 0.42]
Dirks 2017 [48], RET+PS vs RET+PLA-S	1.2	1.28	17	1	1.05	17	16.1%	0.17 [-0.51, 0.84]
Tieland 2012 [58], RET+PS vs RET+PLA-S	1.2	1.07	31	0.3	1.1	31	27.1%	0.82 [0.30, 1.34]
**Subtotal (95% CI)**			**115**			**102**	**100.0%**	**0.28 [0.01, 0.55]**
Heterogeneity: Chi² = 5.64, df = 2 (P = 0.06); I² = 65%								
Test for overall effect: Z = 2.06 (P = 0.04)								
**22.13.2 SPPB, ≥6 mo, <12 mo; Fixed**								
Corcoran 2017 [46], MET+PS vs Con	-0.3	4.2	67	-0.1	3.9	54	55.5%	-0.05 [-0.41, 0.31]
Dirks 2017 [48], RET+PS vs RET+PLA-S	1.5	1.55	17	1.7	1.45	17	15.7%	-0.13 [-0.80, 0.54]
Tieland 2012 [58], RET+PS vs RET+PLA-S	1.4	1.47	31	1.3	1.55	31	28.8%	0.07 [-0.43, 0.56]
**Subtotal (95% CI)**			**115**			**102**	**100.0%**	**-0.03 [-0.30, 0.24]**
Heterogeneity: Chi² = 0.24, df = 2 (P = 0.89); I² = 0%								
Test for overall effect: Z = 0.21 (P = 0.83)								
**22.13.3 SPPB, ≥3 mo, <6 mo; Fixed**								
Corcoran 2017 [46], MET+PS vs Con	0.6	2.9	67	0.4	3.4	54	56.8%	0.06 [-0.30, 0.42]
Dirks 2017 [48], RET+PS vs RET+PLA-S	1.2	1.28	17	1	1.05	17	16.1%	0.17 [-0.51, 0.84]
Tieland 2012 [58], RET+PS vs RET+PLA-S	1.2	1.07	31	0.3	1.1	31	27.1%	0.82 [0.30, 1.34]
**Subtotal (95% CI)**			**115**			**102**	**100.0%**	**0.28 [0.01, 0.55]**
Heterogeneity: Chi² = 5.64, df = 2 (P = 0.06); I² = 65%								
Test for overall effect: Z = 2.06 (P = 0.04)								
**22.13.4 SPPB, <3 mo**								
**Subtotal (95% CI)**			**0**			**0**		**Not estimable**
Heterogeneity: Not applicable								
Test for overall effect: Not applicable								

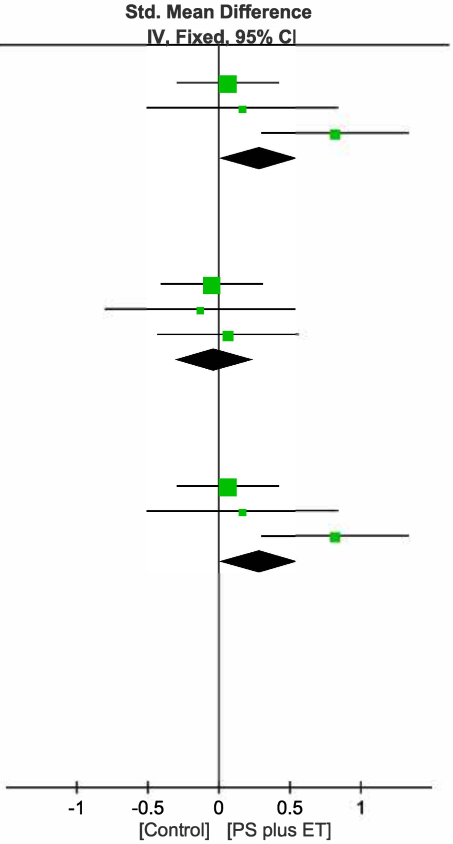

Figure S13. Forest plot summarizing effects of protein supplement (PS) plus exercise training (ET) on SPPB at an overall duration and each follow-up time point. The horizontal line links the lower and upper limits of the 95% CI of this effect. The combined effects are plotted using black diamonds. 95% CI = 95% confidence interval; Fixed = fixed-effects model; Random = random-effects model; Std. = standard; IV = inverse variance; CG = control group; Con = control; MET = multicomponent exercise training; PLA-S, placebo supplement; RET = resistance exercise training; SPPB = short physical performance battery.

Study or Subgroup	PS plus ET Mean	PS plus ET SD	PS plus ET Total	Control Mean	Control SD	Control Total	Weight	Std. Mean Difference IV, Fixed, 95% CI
**22.14.1 Overall follow-up duration; Fixed**								
Beck 2010 [42], RET+PS vs RET	-0.3	9.84	62	-0.4	13.2	59	31.7%	0.01 [-0.35, 0.37]
Chin A Paw 2001 [45], MET+PS vs PLA-S	-0.6	3.4	42	-0.1	3	37	20.5%	-0.15 [-0.60, 0.29]
Ikeda 2016 [52], MET+PS vs MET+PLA-S, Tr1	0.3	15.4	27	0.7	27.6	25	13.6%	-0.02 [-0.56, 0.53]
Ikeda 2016 [52], MET+PS vs MET+PLA-S, Tr2	-0.3	23.7	25	2	32.3	27	13.6%	-0.08 [-0.62, 0.46]
Imaoka 2016 [53], MET+PS vs CG	2.5	37.3	23	-0.14	33	68	18.0%	0.08 [-0.40, 0.55]
Trabal 2015 [59], MET+PS vs MET+PLA-S	14	14.1	7	9	5.6	4	2.6%	0.38 [-0.86, 1.63]
**Subtotal (95% CI)**			**186**			**220**	**100.0%**	**-0.02 [-0.22, 0.18]**
Heterogeneity: Chi² = 0.98, df = 5 (P = 0.96); I² = 0%								
Test for overall effect: Z = 0.18 (P = 0.86)								
**22.14.2 ADL, ≥6 mo, <12 mo**								
Beck 2010 [42], RET+PS vs RET	-0.3	9.84	62	-0.4	13.2	59	100.0%	0.01 [-0.35, 0.37]
**Subtotal (95% CI)**			**62**			**59**	**100.0%**	**0.01 [-0.35, 0.37]**
Heterogeneity: Not applicable								
Test for overall effect: Z = 0.05 (P = 0.96)								
**22.14.3 ADL, ≥3 mo, <6 mo; Fixed**								
Chin A Paw 2001 [45], MET+PS vs PLA-S	-0.6	3.4	42	-0.1	3	37	30.1%	-0.15 [-0.60, 0.29]
Ikeda 2016 [52], MET+PS vs MET+PLA-S, Tr1	0.3	15.4	27	0.7	27.6	25	19.9%	-0.02 [-0.56, 0.53]
Ikeda 2016 [52], MET+PS vs MET+PLA-S, Tr2	-0.3	23.7	25	2	32.3	27	19.9%	-0.08 [-0.62, 0.46]
Imaoka 2016 [53], MET+PS vs CG	2.5	37.3	23	-0.14	33	68	26.3%	0.08 [-0.40, 0.55]
Trabal 2015 [59], MET+PS vs MET+PLA-S	14	14.1	7	9	5.6	4	3.8%	0.38 [-0.86, 1.63]
**Subtotal (95% CI)**			**124**			**161**	**100.0%**	**-0.03 [-0.27, 0.21]**
Heterogeneity: Chi² = 0.95, df = 4 (P = 0.92); I² = 0%								
Test for overall effect: Z = 0.25 (P = 0.80)								
**22.14.4 ADL, <3 mo; Fixed**								
Beck 2010 [42], RET+PS vs RET	-0.01	10.02	62	-0.1	13.44	59	83.6%	0.01 [-0.35, 0.36]
Trabal 2015 [59], MET+PS vs MET+PLA-S	12	18.8	12	8	12.5	12	16.4%	0.24 [-0.56, 1.05]
**Subtotal (95% CI)**			**74**			**71**	**100.0%**	**0.05 [-0.28, 0.37]**
Heterogeneity: Chi² = 0.27, df = 1 (P = 0.60); I² = 0%								
Test for overall effect: Z = 0.28 (P = 0.78)								

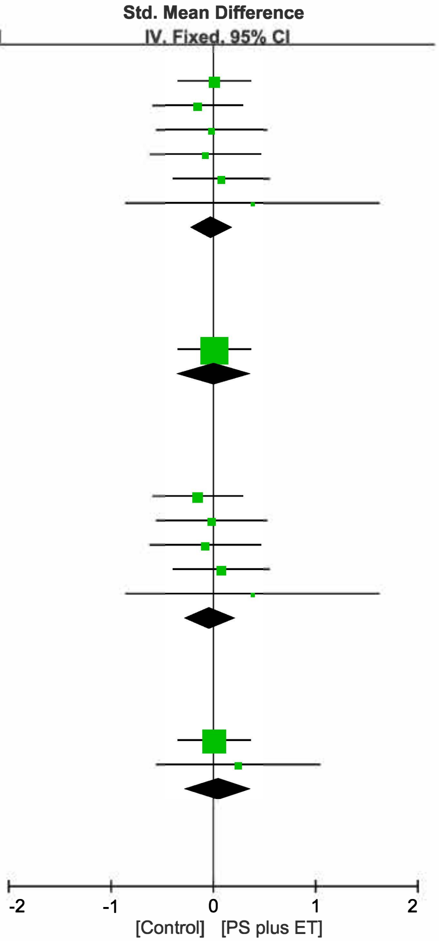

Figure S14. Forest plot summarizing effects of protein supplement (PS) plus exercise training (ET) on activities of daily living (ADL) at an overall duration and each follow-up time point. The horizontal line links the lower and upper limits of the 95% CI of this effect. The combined effects are plotted using black diamonds. 95% CI = 95% confidence interval; Fixed = fixed-effects model; Std. = standard; IV = inverse variance; CG = control group; Con = control; MET = multicomponent exercise training; PLA-S, placebo supplement; RET = resistance exercise training; Tr = treatment session.
